# Antagonism of *Eucalyptus* endophytic fungi against some important crop fungal diseases

**DOI:** 10.3389/fmicb.2025.1523127

**Published:** 2025-02-12

**Authors:** Parmida Aleahmad, Leila Ebrahimi, Naser Safaie, Hassan Reza Etebarian

**Affiliations:** ^1^Department of Entomology and Plant Pathology, Faculty of Agricultural Technology, College of Agriculture and Natural Resources, University of Tehran, Tehran, Iran; ^2^Department of Plant Pathology, Faculty of Agriculture, Tarbiat Modares University, Tehran, Iran

**Keywords:** antagonist, biocontrol, chitinase, cellulase, metabolites, volatile organic compounds

## Abstract

Endophytic fungi colonize plants without causing symptoms, throughout or at least a significant part of their life cycle, forming a plant-fungal association. In this study, endophytic fungi were isolated from *Eucalyptus camaldulensis* trees, and their antifungal activity was evaluated against four significant plant pathogens namely *Botrytis cinerea*, *Fusarium oxysporum* f. sp. *lycopersici*, *Macrophomina phaseolina*, and *Rhizoctonia solani*. For this aim, 754 fungal isolates were obtained from 44 healthy fruit, leaf, and branch samples collected from five provinces of Iran. Subsequently, 27 fungal genera were identified based on morphological characteristics and molecular data of ITS region, with *Neofusicoccum*, *Cladosporium*, *Didymosphaeria*, and *Chaetomium* being the most commonly found genera. Based on the morphological characteristics, 170 isolates were chosen and their antifungal activities were assessed against the aforementioned pathogens *in vitro* through dual culture and volatile organic compounds (VOCs) tests. Based on the results, five isolates comprising *Trichoderma* sp. KL1, *Trichoderma* sp. 8S1, *Chaetomium* sp. DL4, *Phaeophleospora* sp. XL4, and *Pseudosydowia* sp. VL3 were selected for further investigation, which included examining their chitinase and cellulase secretion capabilities as potential antagonism mechanisms and their ability to solubilize phosphate as a growth-promoting mechanism. Furthermore, the antifungal activity of the selected isolates was evaluated against plant pathogens on tomato plants under greenhouse conditions. Their impact on plant growth parameters was also assessed. *In vitro* and greenhouse experiments demonstrated that each selected isolate exhibited varying levels of control against different pathogens. Among the isolates, *Trichoderma* sp. isolates KL1 and 8S1 consistently exhibited the strongest inhibition of disease severity for all four pathogens under greenhouse conditions. Lastly, the selected isolates were identified as *Trichoderma longibrachiatum* KL1, *T. longibrachiatum* 8S1, *Chaetomium globosum* DL4, *Phaeophleospora eucalypticola* XL4, and *Pseudosydowia eucalypti* VL3 based on their morphological features and molecular data of the ITS1-5.8S-ITS2 and *tef-1α* genomic regions.

## 1 Introduction

The widespread occurrence of plant diseases has resulted in substantial economic losses and reduced plant yield and production rates globally, making them a major agricultural concern (Hardoim et al., 2015). Tomato (*Solanum lycopersicum* L.), belonging to the Solanaceae family, ranks as the world’s second most extensively cultivated vegetable crop (Panno et al., 2021). The cultivation of both fresh market and processed tomatoes is hindered by various diseases caused by fungi, bacteria, phytoplasmas, viruses, and viroids. Among these pathogens, *Alternaria solani, Botrytis cinerea, Fusarium oxysporum,* and *Sclerotinia sclerotiorum* stand out as the most critical fungal adversaries (Gilardi et al., 2021). Disease management strategies often rely heavily on the frequent application of fungicides, which can be both expensive and time-consuming (Wenneker and Thomma, 2020). Given the importance of environmental sustainability, public health, and the development of resistance to pathogens, the use of chemical fungicides should be carefully regulated (Latz et al., 2018). Due to these considerations, there is a growing demand for alternative disease management methods. These methods should provide environmentally friendly and economically viable solutions to combat plant diseases (Hardoim et al., 2015; Latz et al., 2018). In addition to selecting disease-resistant plants and implementing other management practices, biocontrol using beneficial microorganisms is increasingly recognized as a crucial component of comprehensive disease management strategies. Recent years have witnessed a heightened effort to identify and utilize these microorganisms (Hardoim et al., 2015; Wani et al., 2015). Furthermore, endophytes represent a promising source of biocontrol agents, as they are well-adapted to colonize and survive within plants without causing significant harm (Zhang et al., 2018; Ebrahimi et al., 2022).

Fungal endophytes colonize inner plant tissues without causing symptoms of disease, thus providing benefits to the host plant while also benefiting from this interaction (Yan et al., 2019). These microorganisms exhibit a broad host range, colonizing diverse plant species and inhabiting all plant organs, encompassing roots, stems, leaves, flowers, fruits, and seeds (Ling et al., 2024). Numerous studies highlight the positive effects of endophytic colonization, including direct benefits like nutrient acquisition and phytohormone production and indirect benefits such as induced resistance, antibiotic and secondary metabolite production, siderophore production, and protection against abiotic and biotic stresses (Baron and Rigobelo, 2022). Endophytic fungi bio-inhibit pathogens through various mechanisms, comprising hyperparasitism, competition, antibiosis, and induced resistance, which can weaken or eliminate them (Latz et al., 2018). These fungi are capable of producing a wide array of chemically diverse secondary metabolites, which exhibit antimicrobial, antifungal, antiparasitic, anticancer, and antiviral activities (Attia et al., 2022). Furthermore, these microorganisms are crucial for their host plants, performing vital functions such as boosting growth and development, augmenting biomass, aiding in water and nutrient uptake, enhancing resistance to different stresses, and facilitating the accumulation of secondary metabolites that confer immunity, allelopathic resistance, and carbon sequestration (Jia et al., 2020). Among the secondary metabolites produced by endophytic fungi are steroids, alkaloids, phenols, isocoumarins, xanthones, quinones, and terpenoids (Manganyi and Ateba, 2020). These fungi can provide benefits through their action as biological control agents and the activation of plant defense responses to biotic stresses (Fontana et al., 2021). Interestingly, endophytic fungi exhibit more diverse metabolic profiles than soil fungi (Hashem et al., 2023). This suggests that endophytes are more likely to provide a consistent and reliable control effect, making them ideal candidates for biocontrol applications (Bardin et al., 2015).

The genus *Eucalyptus* (Myrtaceae), native to Australia and encompassing over 700 species, is globally cultivated for its medicinal, oil, paper, pulp, charcoal, gum, energy, timber, furniture, housing, and other esthetic properties (Labate et al., 2009; Batish et al., 2008). Essential oils derived from various *Eucalyptus* species are extensively utilized across the pharmaceutical, cosmetic, food, and industrial sectors, owing to their abundant chemical makeup and antimicrobial efficacy. Also, essential oils of various *Eucalyptus* species are noted for their diaphoretic, disinfectant, antimalarial, antiseptic, analgesic, anti-inflammatory, antimicrobial, antifungal, antibacterial, and antiviral properties, expectorant, and antioxidant attributes (Marzoug et al., 2011; Ahmad et al., 2023). The *Eucalyptus* oil is a complex mixture of a variety of monoterpenes and sesquiterpenes, and aromatic phenols, oxides, ethers, alcohols, esters, aldehydes and ketones which possess toxicity against a wide range of microbes including bacteria and fungi (both soil-borne and post-harvest pathogens), insects and nematodes (Batish et al., 2008). Interestingly, fungal endophytes are prolific producers of compounds with practical applications in agrochemicals or medicine such as antiviral, antimicrobial, anticancer, immunosuppressive, antidiabetic, antioxidant, etc. which may be similar to those produced by their host plants (Fadiji and Babalola, 2020).

Various studies have been conducted to evaluate the *Eucalyptus* endophytic fungi. Mao et al. (2021) obtained 80 endophytic fungal isolates from *E. exserta*, identifying 13 genera: *Penicillium* Link, *Chaetomium* Kunze, *Cladosporium* Link, *Phyllosticta* Pers., *Eutypella* (Nitschke) Sacc., *Purpureocillium* Luangsa-ard, Houbraken and Samson, *Gongronella* Ribaldi, *Talaromyces* C.R. Benj., *Pestalotiopsis* Steyaert, *Fusarium* Link, *Lophiostoma* Ces. and De Not., *Scedosporium* Sacc. ex Castell. and Chalm., and *Pseudallescheria* Negr. and I. Fisch.

Despite extensive research on endophytic fungi in various plant species, their association with *Eucalyptus* trees in Iran as well as their antagonistic effects against plant pathogens remain poorly understood. This investigation aimed to isolate and characterize endophytic fungi from *E. camaldulensis* Dehnh. in some provinces of Iran, and evaluate their potential to control some important plant pathogens, including *B. cinerea*, *F. oxysporum* f. sp. *lycopersici* race 3, *Macrophomina phaseolina*, and *Rhizoctonia solani in vitro* and in greenhouse.

## 2 Materials and methods

### 2.1 Sample collection and endophytic fungi isolation

Healthy samples of *E. camaldulensis*, including leaves, branches, and fruits, were collected from Tehran, Alborz, Qom, Isfahan, and Mazandaran provinces of Iran, during autumn of 2022. After cleaning with tap water, samples underwent sterilization using a method established by Strobel and Daisy (2003), which involved treatments with ethanol, sodium hypochlorite, and sterile water. For branches, the outer layer was removed under sterile conditions. Samples were cut and incubated on water agar medium at 25°C for 2 to 4 weeks. Emerging fungi were transferred onto potato dextrose agar (PDA) for purification. Obtained fungal isolates were identified at the genus level based on their morphological characteristics using authentic mycological monographs and literatures prepared by Simmons (2007), Ellis (1971, 1976), Klich and Pitt (1988), Klich (2002), Sivanesan (1987), and Watanabe (2002). Purified isolates were preserved at 4°C, and archived at the Fungal Collection of Agricultural Biotechnology Research Institute in Karaj, Iran.

### 2.2 Biocontrol experiments *in vitro*

#### 2.2.1 Dual culture method

Initially, the antifungal activity of 170 endophytic isolates was evaluated against four phytopathogenic fungi including *B. cinerea* (Jalali et al., 2021), *F. oxysporum* f.sp. *lycopersici* race 3, *M. phaseolina* (Shirali, 2017), and *R. solani* by the triple spot culture test. Subsequently, antagonistic isolates were selected for a dual culture test as described by Dennis and Webster (1971). The plates were incubated at 25°C for 5 days. The growth inhibition is calculated using the formula GI% = [(a − b)/a] × 100, where ‘GI’ is the percentage of growth inhibition (GI), ‘a’ is the pathogen’s colony diameter in control, and ‘b’ is the pathogen’s colony diameter in dual culture (Etebarian et al., 2005). The experiment was conducted with three replicates and repeated twice.

#### 2.2.2 Volatile organic compound-mediated interactions

Further investigation focused on VOCs emitted by endophytes, according to Lillbro (2005). This involved growing both endophytes and pathogens on PDA, layering the pathogen-containing plates atop those with endophytes, sealing them with Parafilm to trap VOCs, and incubating at 25°C. Furthermore, control treatments consisted of plates with pathogens placed between uninoculated PDA plates. After 1 week, pathogen growth was assessed, and the abovementioned formula was applied to quantify reductions. The experiment was carried out with three replicates and repeated twice.

#### 2.2.3 Chitinase activity

The method for assessing chitinase production followed Hsu and Lockwood (1975). Endophytic isolates were cultured on chitin agar (0.4% colloidal chitin +1.5% agar, pH 7.2), prepared according to Berger and Reynold (1958). After 5 days at 25°C, chitinase activity was indicated by a clear zone around the colonies, quantified by comparing the diameter of the clear zone to the colony diameter. The experiment was carried out with three replicates and repeated twice.

#### 2.2.4 Cellulase activity

Fungal isolates were grown on a CMC agar medium (containing KH_2_PO_4_, CaCl_2_, FeSO_4_·7H_2_O, CMC, and agar, pH 7.2) at 25°C for 7 days. For evaluating the cellulase activity, the medium was first stained with Congo Red solution for 20 min, then rinsed with NaCl for 15 min. Cellulase activity was calculated as the ratio of clear zone diameter to colony diameter, aligning with Majidi et al. (2011) method. The experiment was performed in triplicate and repeated twice.

#### 2.2.5 Phosphate solubilization

Endophytic isolates were tested for their ability to solubilize phosphate by culturing them on Sperber medium (insoluble phosphate-enriched, pH 7.2) at 25°C for 7 days. The solubilization index was calculated by comparing the diameter of the clear zone around each colony to the colony’s diameter, following Sperber (1958) method. The experiment was conducted in triplicate and repeated twice.

### 2.3 Greenhouse experiments

#### 2.3.1 Plant cultivation

Tomato (cv. Early Urbana Y) seeds were sterilized in a 0.5% sodium hypochlorite solution (with 5% active chlorine) for 5 min, followed by 3 sterile water rinses, as per Herrera-Téllez et al. (2019). Seeds were germinated in a sterile perlite-coco peat mixture in seedling trays, and kept in a greenhouse for 3 weeks. Plants were grown in 14-inch plastic pots filled with a custom soil mix of sterilized field soil, coco peat, and sand, autoclaved 3 times to ensure sterility. Two seedlings per pot were planted to provide optimal growing conditions.

#### 2.3.2 Biocontrol of grey mold disease

An initial pathogenicity test was conducted to assess the pathogenicity of *B. cinerea* B2, and optimize greenhouse conditions. For the biocontrol assay, tomato plants were inoculated with *B. cinerea* at a concentration of 10^5^ conidia/ml using a spray method, targeting the aerial parts of the plants. This inoculation occurred 1 week after the plants had been treated with endophytic fungi at 10^8^ spores/ml, also applied with the same method. Control treatment received sterile distilled water. Plants were treated at the 4–6 leaf stage. After inoculation, each pot was sealed with a plastic bag to maintain 100% humidity for 48 h, then moved to a greenhouse with >80% humidity and 20 ± 2°C. Observations were daily made to track symptom development. Disease severity was measured by quantifying affected areas and calculating infection percentages. The disease inhibition rate of each antagonist was compared to the control to determine efficacy (Ebrahimi et al., 2023). Three pots each containing two seedlings were considered for each treatment and the experiment was repeated twice.

#### 2.3.3 Biocontrol of Fusarium wilt

An initial pathogenicity test was performed to evaluate the virulence of *F. oxysporum* f. sp. *lycopersici* race 3 (FOL). For biocontrol test, tomato seedlings were inoculated with fungal endophytes using a 30 min root-dip method at a concentration of 10^8^ conidia/ml. One week later, seedlings were dipped in a FOL suspension of 5 × 10^7^ conidia/ml (Abbasi et al., 2019). Control seedlings were dipped in sterile distilled water. Seedlings were watered daily, kept at 28 ± 2°C, and exposed to 16 h of light and 8 h of darkness. Disease severity was monitored daily and scored using a scale from 0 to 5, where 0% indicates no symptoms, and 100% indicates death (Marlatt et al., 1996). Each treatment involved three pots, each with two seedlings, and the entire experiment was duplicated.

#### 2.3.4 Biocontrol of charcoal rot disease

An initial pathogenicity test confirmed the pathogenicity of *M. phaseolina* M14. Isolates of endophytic fungi and *M. phaseolina* were grown on PDA for 7 days, then transferred on autoclaved sand-corn meal medium in Erlenmeyer flasks and incubated at 25°C for endophytes and 30°C for *M. phaseolina* for 3 weeks (Etebarian et al., 2007). Inoculums were mixed with potting mix at a 10% ratio by weight for both endophytes and *M. phaseolina*, with a one-week interval before planting (Jimenez-Diaz et al., 1983). Two seeds per pot were sown in each pot. Plants were grown in a greenhouse at 30 ± 2°C. Symptoms were monitored daily, and disease severity was rated from 1 to 5 based on discoloration and microsclerotia visibility (Paris et al., 2006). For every treatment, three pots were utilized, and the experimental procedures were carried out twice.

#### 2.3.5 Biocontrol of Rhizoctonia damping-off disease

Following the initial pathogenicity tests, fungal isolates’ inoculum was prepared by soaking corn seeds in distilled water, autoclaving them, and inoculating with *R. solani* 124 and endophytic isolates. All inoculums were incubated for 6 weeks at 25°C in darkness. As a control, autoclaved corn seeds were also incubated similarly. An autoclaved soil mixture (soil: perlite: peat moss, 3:1:1) was prepared and mixed with the inoculum (fungi-inoculated corn seeds, 8 g/kg soil). Tomato seedlings were planted in this inoculated soil 1 week after endophyte treatment, and the pots were incubated in a greenhouse with 40% humidity, 22 ± 2°C, and a 16 h light cycle. Watering was done daily, and symptoms were monitored daily. After 3 weeks, data were collected on lesion occurrence, and root rot ratings using a scale from 1 (no root rot) to 5 (pre-emergence damping-off and minimal roots) (Dorrance et al., 2003). In the setup for each treatment, 3 pots were prepared, each with 2 seedlings, and the experiment was conducted in duplicate.

#### 2.3.6 Estimating plant growth parameters

After completing the experiments (3 weeks after inoculation), plant growth parameters including fresh and dry weight as well as plant height were evaluated. Fresh and dry weights were measured using an analytical balance, with plants dried at 60°C for 4 h before weighing. All measurements were recorded for 6 plants of each treatment with two independent repetitions.

### 2.4 Evaluation of endophytic isolate colonization

For this purpose, tomato plants treated with endophyte isolates were cleaned under tap water to remove soil. Plant material was then disinfected using the method described by Strobel and Daisy (2003). Fungi were cultured from leaves, stems, and roots onto WA medium, purified using the hyphal tip method. The presence of inoculated fungi was confirmed through their colonization of various plant parts. Fungal isolates were morphologically identified, following method used by Ebrahimi et al. (2022).

### 2.5 Statistical analysis

The experiments were conducted in a completely randomized design and complete randomized block design for *in vitro* and in greenhouse investigations, respectively. Statistical analysis was carried out using SAS software, version 9.0. Initially, it was verified that the data conformed to a normal distribution. Subsequently, the data underwent analysis of variance (ANOVA), followed by Duncan’s multiple range test (Steel and Torrie, 1980).

### 2.6 Molecular identification of endophytic fungi

Antagonistic endophytic fungi were identified based on morphological features on Oatmeal Agar (OA), Potato Carrot Agar (PCA), Malt Extract Agar (MEA), and PNA (Pine Needle Powder Agar) media, with emphasis on colony characteristics, mycelia structure, teleomorphic/anamorphic stages, and reproductive structures, using various monographs including Samuels (2006), Samuels et al. (2012), Crous et al. (2016), Thambugala et al. (2014), Prokhorov and Linnik (2011).

Selected isolates showing antagonism against pathogenic fungi were cultured on PDA under dark conditions at 25°C for 7 days. DNA was extracted using the CTAB method (Carter-House, 2020), and then amplified for ITS and *tef-1α* regions using ITS1/ITS4 (White et al., 1990), and EF1/EF2 primers (O'Donnell et al., 1998), following PCR conditions from Ebrahimi and Fotouhifar (2016). DNA sequencing was done at Noor Genetics Center, Tehran, Iran.

The sequences obtained were compared to related species using NCBI BLAST to confirm the taxonomy. Sequences were submitted to GenBank for public access. In the phylogenetic analyses, genomic region sequences of *tef-1α* and ITS from various species were aligned with corresponding reference sequences of related species, retrieved from GenBank (Supplementary Table S1), using ClustalW (Thompson et al., 1994). Subsequently, Maximum Likelihood (ML) analysis (Felsenstein, 1981) was executed through a heuristic search, facilitated by MEGA 10.2 (Takeuchi et al., 2018).

## 3 Results

### 3.1 Endophytic fungal isolates

**Figure 1:**
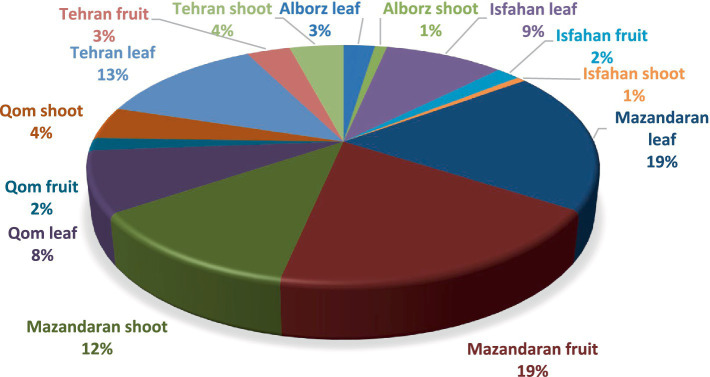
The relative abundance (%) of *Eucalyptus* fungal endophytes isolated from different tissues and regions.

From 44 healthy *Eucalyptus* leaf, fruit and branch samples, a total of 754 endophytic fungal isolates were obtained, including 389 isolates from leaves, 197 from fruits, and 168 from branches (Figure 1). One hundred seventy isolates were subsequently selected as representatives, with 95 isolates from leaves, 47 from branches, and 28 from fruits, based on their morphological characteristics and growth rates for biocontrol assays.

Overall, 27 fungal genera were identified through their morphological features, including *Alternaria, Aspergillus, Bipolaris, Botrytis, Chaetomium, Cladosporium, Coniosporium, Cytospora, Didymella, Didymosphaeria, Fusarium, Gymnoascus, Hyalocylindrophora, Iodophanus, Microsphaeropsis, Neofusicoccum, Neoschizothecium, Niesslia, Paecilomyces, Penicillium, Pestalotiopsis, Peziza, Phaeophleospora, Pseudosydowia, Rhizopus, Trichoderma* and *Ulocladium*. Based on the results, the most frequently isolated endophytes from fruits, leaves, and branches belonged to the *Neofusicoccum*, *Cladosporium*, and *Chaetomium* genera, respectively.

### 3.2 Screening of endophytic fungi for antifungal activity

Based on the results of the primary triple spot culture test, 50 isolates exhibited antagonistic effects against all four pathogens (Supplementary Table S2). In the following stage, five isolates with the highest inhibitory effect were selected for the dual culture test.

#### 3.2.1 Dual culture test

**Figure 2:**
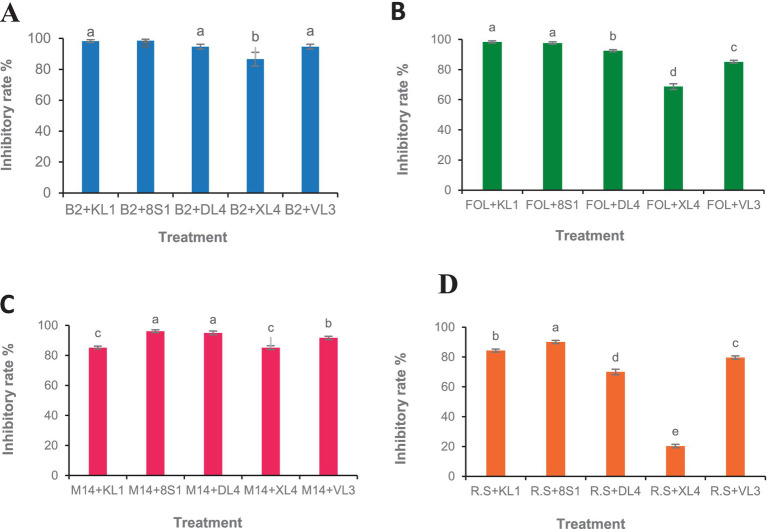
Growth inhibition percent of fungal pathogens [**(A)** B2: *B. cinerea* B2, **(B)** FOL: *F. oxysporum* f. sp. *lycopersici*, **(C)** M14: *M. phaseolina* M14, **(D)** R.S: *R. solani* 124] by endophytic fungi (KL1: *Trichoderma* sp. KL1, 8S1: *Trichoderma* sp. 8S1, DL4: *Chaetomium* sp. DL4, XL4: *Phaeophleospora* sp. XL4, and VL3: *Pseudosydowia* sp. VL3) in dual culture test. Data are presented as means ± standard error (SE) based on three replicates. According to Duncan’s multiple range test, values with different letters indicate statistically significant differences (*p* ≤ 0.05).

**Figure 3:**
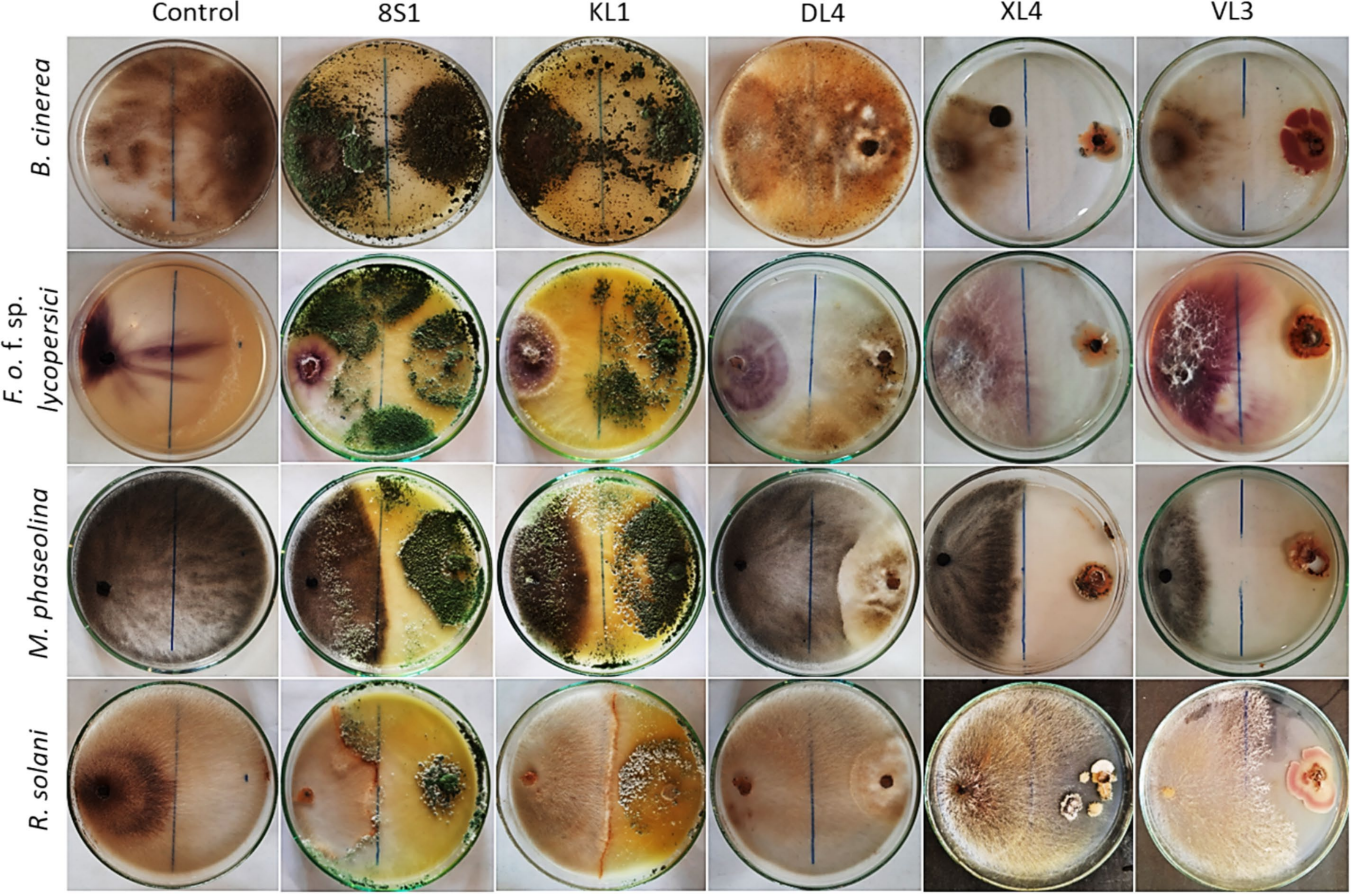
Inhibition of pathogens mycelia growth after 7 days in dual culture test. KL1: *Trichoderma* sp. KL1, 8S1: *Trichoderma* sp. 8S1, DL4: *Chaetomium* sp. DL4, XL4: *Phaeophleospora* sp. XL4, and VL3: *Pseudosydowia* sp. VL3.

Five selected isolates exhibited potential activity against *B. cinerea* B2, *F. oxysporum* f. sp. *lycopersici*, *M. phaseolina* M14 and *R. solani* 124. Selected endophytic isolates, namely *Trichoderma* sp. 8S1, *Trichoderma* sp. KL1, *Chaetomium* sp. DL4, *Phaeophleospora* sp. XL4, and *Pseudosydowia* sp. VL3 showed different inhibitory rates (%) against each pathogen, and both *Trichoderma* sp. isolates displayed the highest inhibition against the mycelia growth of the pathogens (Figures 2, 3).

#### 3.2.2 Volatile organic compound-mediated interactions

**Figure 4:**
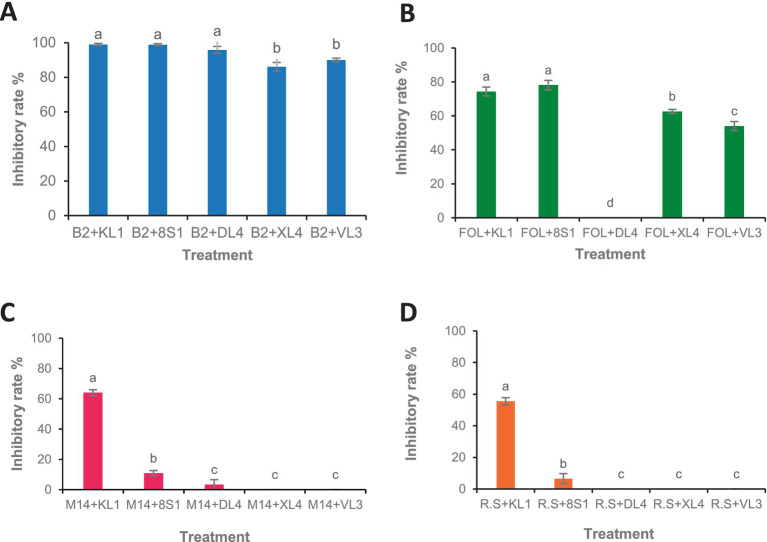
Growth inhibition percent of fungal pathogens [**(A)** B2: *B. cinerea* B2, **(B)** FOL: *F. oxysporum* f. sp. *lycopersici*, (C) M14: *M. phaseolina* M14, **(D)** R.S: *R. solani* 124] by endophytic fungi (KL1: *Trichoderma* sp. KL1, 8S1: *Trichoderma* sp. 8S1, DL4: *Chaetomium* sp. DL4, XL4: *Phaeophleospora* sp. XL4, and VL3: *Pseudosydowia* sp. VL3) in volatile organic compound test. Data are presented as means ± standard error (SE) based on three replicates. According to Duncan’s multiple range test, values with different letters indicate statistically significant differences (*p* ≤ 0.05).

**Figure 5:**
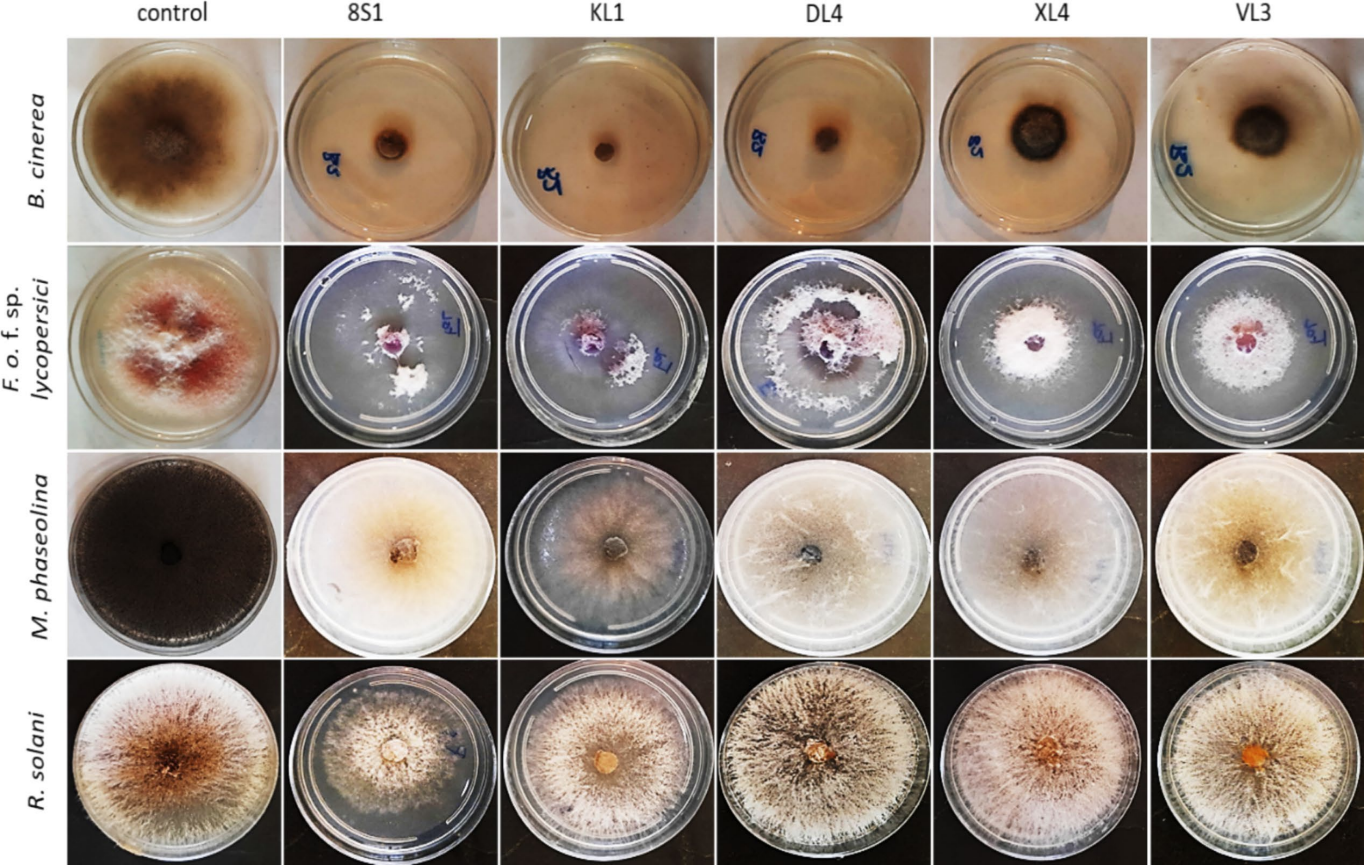
Inhibition of mycelia growth of fungal pathogens after 7 days in volatile organic compounds tests. KL1: *Trichoderma* sp. KL1, 8S1: *Trichoderma* sp. 8S1, DL4: *Chaetomium* sp. DL4, XL4: *Phaeophleospora* sp. XL4, and VL3: *Pseudosydowia* sp. VL3.

According to the results, the VOCs produced by all antagonists inhibited the growth of *B. cinerea* mycelia with the isolate *Trichoderma* sp. KL1 by more than 80% exhibits the highest inhibition. Except *Chaetomium* sp. DL4, all other isolates showed inhibitory effects on the mycelial growth of *Fusarium oxysporum* f. sp. *lycopersici*. Additionally, both *Trichoderma* sp. isolates exhibited the strongest inhibition, exceeding 70%. In contrast, *Pseudosydowia* sp. VL3 displayed the lowest inhibitory activity, with approximately 54% inhibition against *Fusarium oxysporum* f. sp. *lycopersici*. Among the five investigated isolates, only *Trichoderma* sp. KL1 produced VOCs that effectively inhibited the mycelial growth of *M. phaseolina* M14 (60%) and *R. solani* 124 (55%). Other isolates showed no inhibition against these two pathogens. In particular, *Trichoderma* sp. KL1 reduced *M. phaseolina* microsclerotia formation (Figures 4, 5).

#### 3.2.3 Enzyme activity and phosphate solubilization

Based on the results of cellulase secretion, all isolates except *Pseudosydowia* sp. VL3 showed cellulase activity. Similarly, the capacity for chitinase secretion was observed among all 5 isolates, as evidenced by the presence of a transparent halo surrounding the colonies (Table 1).

**Table 1:** Enzyme activity and phosphate solubilization of selected endophytic fungal isolates.

Isolates	Cellulase	Chitinase	Phosphate solubilization
*Trichoderma* sp. KL1	3.19 ± 0.84^*^	1.89 ± 0.80	1.29 ± 0.11
*Trichoderma* sp. 8S1	3.48 ± 0.95	2.79 ± 0.35	1.27 ± 0.05
*Chaetomium* sp. DL4	1.33 ± 0.08	1.84 ± 0.56	1.31 ± 0.11
*Phaeophleospora* sp. XL4	1.41 ± 0.49	2.04 ± 0.49	0.00 ± 0.00
*Pseudosydowia* sp. VL3	0.00 ± 0.00	1.71 ± 0.53	0.00 ± 0.00

^*^The mean of halo zone diameter/ colony diameter ± standard deviation.

The results also revealed that only isolates *Trichoderma* sp. 8S1, *Trichoderma* sp. KL1, and *Chaetomium* sp. DL4 demonstrated the capability to decompose phosphate and concurrently produce a transparent halo (Table 1).

### 3.3 Biocontrol assays under greenhouse conditions

**Figure 6:**
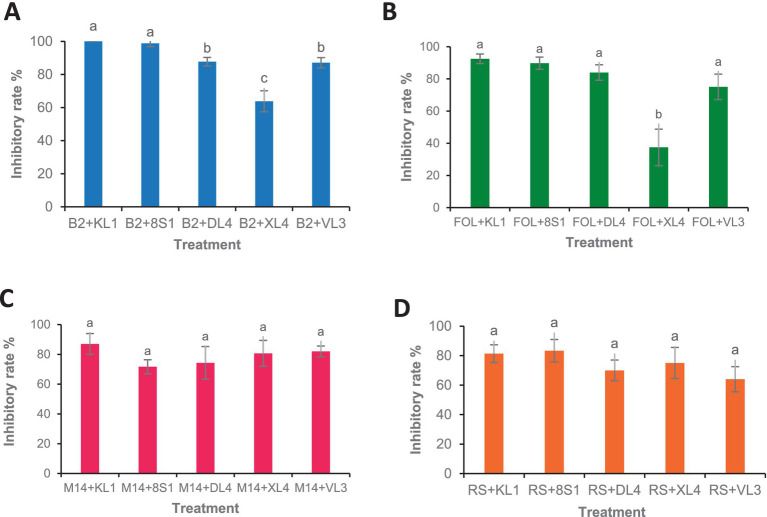
Antagonistic effect of endophytic fungi (KL1: *Trichoderma* sp. KL1, 8S1: *Trichoderma* sp. 8S1, DL4: *Chaetomium* sp. DL4, XL4: *Phaeophleospora* sp. XL4, and VL3: *Pseudosydowia* sp. VL3.) against pathogenic isolates [**(A)** B2: *B. cinerea* B2, **(B)** FOL: *F. oxysporum* f. sp. *lycopersici*, **(C)** M14: *M. phaseolina* M14, **(D)** R.S: *R. solani* 124] in biocontrol tests on tomato plants under greenhouse conditions. Data are presented as means ± standard error (SE) based on six replicates. According to Duncan’s multiple range test, values with different letters indicate statistically significant differences (*p* ≤ 0.05).

**Figure 7:**
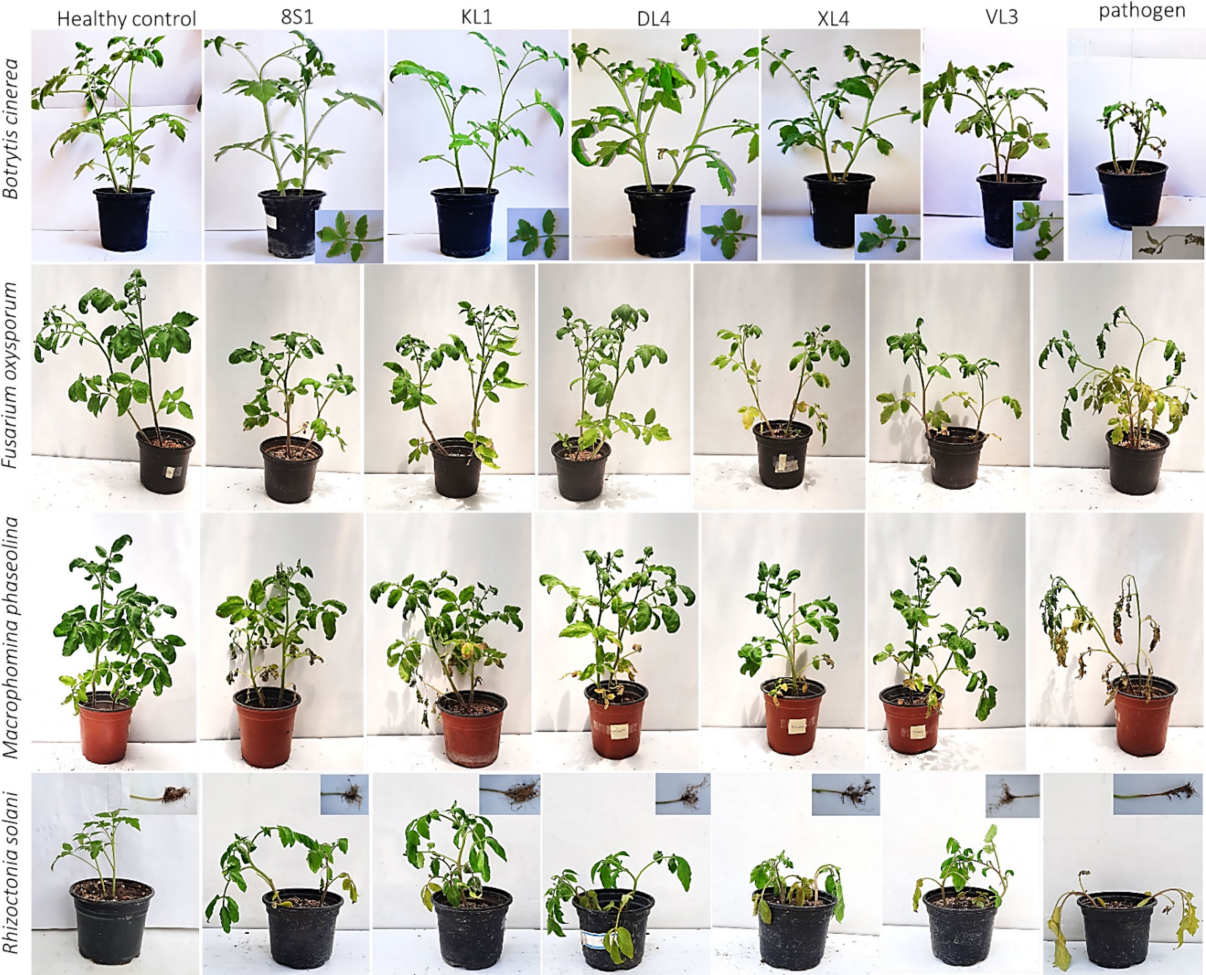
Effect of endophyte isolates (KL1: *Trichoderma* sp. KL1, 8S1: *Trichoderma* sp. 8S1, DL4: *Chaetomium* sp. DL4, XL4: *Phaeophleospora* sp. XL4, and VL3: *Pseudosydowia* sp. VL3) on development of symptoms caused by pathogens (*B. cinerea* B2, *F. oxysporum* f. sp. *lycopersici*, *M. phaseolina* M14, and *R. solani* 124) on tomato plants under greenhouse conditions.

The results of greenhouse tests showed that each endophytic isolate possesses a distinct and remarkable capability in controlling each pathogen. Isolate *Trichoderma* sp. KL1 consistently demonstrated the strongest inhibitory effects against *B. cinerea*, *F. oxysporum* f. sp. *lycopersici*, and *M. phaseolina*. Conversely, *Trichoderma* sp. 8S1 exhibited the highest inhibition against *R. solani*. Additionally, *Phaeophleospora* sp. XL4 displayed the lowest inhibitory activity against *B. cinerea* and *F. oxysporum* f. sp. *lycopersici*, while *Trichoderma* sp. 8S1 and *Pseudosydowia* sp. VL3 showed the lowest inhibition against *M. phaseolina* and *R. solani*, respectively (Figures 6, 7).

### 3.4 The endophytic fungi effect on the growth indices of tomato plants

Plants inoculated exclusively with endophytic fungi did not exhibit any substantial variation in terms of fresh weight, dry weight, and the height of aerial part in comparison to their healthy treatments. On the other hand, a notable disparity was observed among the growth indices of plants treated with endophytic fungi and pathogens, in comparison to those plants that had been treated only with pathogens. It is remarkable that the decline in these indices was less pronounced relative to the healthy controls (Tables 2–5).

**Table 2:** The average fresh weight (g), dry weight (g), and height (cm) of tomato plants inoculated with *B. cinerea* B2 and treated with endophytic fungi in the pot experiment.

Treatments	Heights (cm)	Fresh weight (g)	Dry weight (g)
B2 + KL1	33.54 ± 2.81 a	9.99 ± 0.87 abcd	1.54 ± 0.31 ab
B2 + 8S1	33.10 ± 3.38 a	9.44 ± 0.99 abcde	1.11 ± 0.21 bc
B2 + DL4	28.58 ± 3.56 a	8.01 ± 1.28 cdef	0.75 ± 0.17 bc
B2 + XL4	27.63 ± 3.75 a	7.65 ± 1.47 fe	0.70 ± 0.11 bc
B2 + VL3	29.35 ± 3.16 a	10.31 ± 1.83 abc	0.90 ± 0.16 bc
KL1	34.81 ± 1.93 a	10.18 ± 1.13 abcd	1.75 ± 0.44 a
8S1	33.44 ± 2.98 a	10.05 ± 1.04 abcd	1.76 ± 0.22 a
DL4	31.53 ± 3.42 a	9.36 ± 0.91 abcde	1.15 ± 0.23 bc
XL4	29.78 ± 3.79 a	8.76 ± 1.27 bcde	0.80 ± 0.13 bc
VL3	30.43 ± 3.98 a	10.42 ± 1.58 ab	0.93 ± 0.18 abc
B2	14.80 ± 3.37 b	6.26 ± 1.28 f	0.50 ± 0.15 d
Control	23.00 ± 2.99 a	8.46 ± 1.44 bcdf	0.77 ± 0.17 bc

Data are presented as means ± standard error (SE) based on six replicates (three pots with two plants each). Means with different letters in each column are significantly different according to Duncan’s multiple range test (*p* ≤ 0.05). B2: *B. cinerea* B2, KL1: *Trichoderma* sp. KL1, 8S1: *Trichoderma* sp. 8S1, DL4: *Chaetomium* sp. DL4, XL4: *Phaeophleospora* sp. XL4, and VL3: *Pseudosydowia* sp. VL3.

**Table 3:** The average fresh weight (g), dry weight (g), and height (cm) of tomato plants inoculated with *F. oxysporum* f. sp. *lycopersici* race 3, and treated with endophytic fungi in the pot experiment.

Treatments	Aerial fresh weight (g)	Root fresh weight (g)	Aerial dry weight (g)	Root fresh weight (g)	Height (cm)
FOL + KL1	7.63 ± 1.08 a	1.30 ± 023 bcd	1.02 ± 0.48 ab	0.42 ± 0.31 ab	33.67 ± 1.37 a
FOL + 8S1	8.21 ± 1.06 a	1.17 ± 0.31 cd	1.12 ± 0.15 a	0.65 ± 0.18 ab	34.50 ± 2.26 a
FOL + DL4	7.85 ± 0.96 a	1.20 ± 0.42 bcd	1.11 ± 0.26 a	0.50 ± 0.15 ab	34.33 ± 1.97 a
FOL + XL4	7.74 ± 0.98 a	1.19 ± 0.37 bcd	1.29 ± 0.34 a	0.60 ± 0.24 ab	34.17 ± 2.14 a
FOL + VL3	8.68 ± 1.44 a	1.21 ± 0.24 bcd	1.26 ± 0.20 a	0.50 ± 0.18 ab	34.83 ± 2.93 a
KL1	8.00 ± 0.93 a	1.28 ± 0.31 bcd	1.25 ± 0.40 a	0.57 ± 0.20 ab	34.50 ± 2.23 a
8S1	8.80 ± 1.06 a	1.19 ± 0.27 bcd	1.24 ± 0.47 a	0.68 ± 0.23 a	34.67 ± 2.42 a
DL4	8.16 ± 0.78 a	1.22 ± 0.34 bcd	1.40 ± 0.34 a	0.65 ± 0.16 ab	34.50 ± 2.26 a
XL4	8.71 ± 0.88 a	1.20 ± 0.34 bcd	1.27 ± 0.50 a	0.62 ± 0.26 ab	34.67 ± 1.37 a
VL3	8.62 ± 1.15 a	1.23 ± 0.36 bcd	1.20 ± 0.30 a	0.58 ± 0.25 ab	33.67 ± 1.86 a
FOL	6.02 ± 1.73 b	0.77 ± 0.28 d	0.59 ± 0.15 b	0.30 ± 0.11 ab	33.00 ± 1.79 a
Control	8.32 ± 0.81 a	1.32 ± 0.32 bcd	1.26 ± 0.25 a	0.51 ± 0.21 ab	33.83 ± 2.14 a

Data are presented as means ± standard error (SE) based on six replicates (three pots with two plants each). Means with different letters in each column are significantly different according to Duncan’s multiple range test (*p* ≤ 0.05). FOL: *F. oxysporum f.* sp. *lycopersici,* KL1: *Trichoderma* sp. KL1, 8S1: *Trichoderma* sp. 8S1, DL4: *Chaetomium* sp. DL4, XL4: *Phaeophleospora* sp. XL4, and VL3: *Pseudosydowia* sp. VL3.

**Table 4:** The average fresh weight (g), dry weight (g), and height (cm) of tomato plants inoculated with *M. phaseolina* M14, and treated with endophytic fungi in the pot experiment.

Treatments	Aerial fresh weight (g)	Root fresh weight (g)	Aerial dry weight (g)	Root fresh weight (g)	Height (cm)
M14 + KL1	8.22 ± 0.92 a	1.45 ± 0.32 a	1.16 ± 0.33 a	0.53 ± 0.20 a	33.17 ± 1.72 a
M14 + 8S1	8.16 ± 0.95 a	1.50 ± 0.19 a	1.25 ± 0.50 a	0.59 ± 0.11 a	34.00 ± 1.67 a
M14 + DL4	8.29 ± 0.48 a	1.39 ± 0.33 a	1.45 ± 0.37 a	0.56 ± 0.10 a	34.00 ± 2.37 a
M14 + XL4	8.38 ± 0.84 a	1.32 ± 0.17 a	1.20 ± 0.30 a	0.58 ± 0.13 a	34.33 ± 1.63 a
M14 + VL3	8.50 ± 0.83 a	1.35 ± 0.27 a	1.20 ± 0.65 a	0.57 ± 0.19 a	33.50 ± 1.87 a
KL1	8.35 ± 1.01 a	1.54 ± 0.21 a	1.66 ± 0.22 a	0.54 ± 0.19 a	34.17 ± 2.23 a
8S1	8.24 ± 0.96 a	1.55 ± 0.25 a	1.33 ± 0.40 a	0.64 ± 0.13 a	34.17 ± 2.56 a
DL4	8.29 ± 1.01 a	1.39 ± 0.24 a	1.68 ± 0.76 a	0.61 ± 0.07 a	34.33 ± 2.42 a
XL4	8.35 ± 1.02 a	1.37 ± 0.43 a	1.50 ± 0.27 a	0.58 ± 0.10 a	34.50 ± 1.87 a
VL3	8.94 ± 1.48 a	1.42 ± 0.28 a	1.49 ± 0.88 a	0.58 ± 0.15 a	34.67 ± 1.97 a
M14	6.23 ± 0.83 b	0.78 ± 0.20 b	0.80 ± 0.15 b	0.44 ± 0.10 a	32.83 ± 1.17 a
Control	8.58 ± 0.78 a	1.37 ± 0.24 a	1.37 ± 0.24 a	0.56 ± 0.28 a	34.17 ± 1.72 a

Data are presented as means ± standard error (SE) based on six replicates (three pots with two plants each). Means with different letters in each column are significantly different according to Duncan’s multiple range test (*p* ≤ 0.05). M14: *M. phaseolina* M14, KL1: *Trichoderma* sp. KL1, 8S1: *Trichoderma* sp. 8S1, DL4: *Chaetomium* sp. DL4, XL4: *Phaeophleospora* sp. XL4, and VL3: *Pseudosydowia* sp. VL3.

**Table 5:** The average fresh weight (g), dry weight (g), and height (cm) of tomato plants inoculated with *R. solani* 124, and treated with endophytic fungi in the pot experiment.

Treatments	Aerial fresh weight (g)	Aerial fresh weight (g)	Root fresh weight (g)	Aerial dry weight (g)	Root fresh weight (g)	Height (cm)
R.S + KL1	2.53 ± 0.56 a	2.53 ± 0.56 a	0.66 ± 0.09 a	0.87 ± 0.16 a	0.330 ± 0.09 ab	23.17 ± 1.94 a
R.S + 8S1	2.42 ± 0.61 a	2.42 ± 0.61 a	0.64 ± 0.13 a	0.82 ± 0.19 a	0.35 ± 0.09 ab	22.83 ± 1.47 a
R.S + DL4	2.36 ± 0.54 a	2.36 ± 0.54 a	0.60 ± 0.05 a	0.81 ± 0.17 a	0.31 ± 0.05 ab	21.83 ± 1.47 a
R.S + XL4	2.28 ± 0.40 a	2.28 ± 0.40 a	0.64 ± 0.08 a	0.77 ± 0.13 a	0.30 ± 0.05 ab	22.33 ± 1.63 a
R.S + VL3	2.12 ± 0.33 a	2.12 ± 0.33 a	0.60 ± 0.06 a	0.73 ± 0.10 a	0.29 ± 0.04 ab	22.50 ± 1.52 a
KL1	2.57 ± 0.45 a	2.57 ± 0.45 a	0.70 ± 0.12 a	0.89 ± 0.13 a	0.37 ± 0.08 a	24.00 ± 2.53 a
8S1	2.60 ± 0.49 a	2.60 ± 0.49 a	0.70 ± 0.19 a	0.88 ± 0.16 a	0.37 ± 0.11 a	23.67 ± 1.37 a
DL4	2.43 ± 0.58 a	2.43 ± 0.58 a	0.60 ± 0.08 a	0.84 ± 0.18 a	0.33 ± 0.09 ab	22.67 ± 2.34 a
XL4	2.44 ± 0.51 a	2.44 ± 0.51 a	0.70 ± 0.10 a	0.82 ± 0.16 a	0.32 ± 0.07 ab	23.00 ± 2.00 a
VL3	2.42 ± 0.41 a	2.42 ± 0.41 a	0.64 ± 0.06 a	0.82 ± 0.13 a	0.32 ± 0.05 ab	22.33 ± 1.63 a
R.S	1.99 ± 0.18 a	1.99 ± 0.18 a	0.51 ± 0.08 a	0.68 ± 0.05 a	0.23 ± 0.04 b	21.00 ± 1.41 a
Control	2.11 ± 0.23 a	2.11 ± 0.23 a	0.57 ± 0.08 a	0.72 ± 0.08 a	0.29 ± 0.04 ab	22.33 ± 1.51 a

Data are presented as means ± standard error (SE) based on six replicates (three pots with two plants each). Means with different letters in each column are significantly different according to Duncan’s multiple range test (*p* ≤ 0.05). R.S: *R. solani* 124, KL1: *Trichoderma* sp. KL1, 8S1: *Trichoderma* sp. 8S1, DL4: *Chaetomium* sp. DL4, XL4: *Phaeophleospora* sp. XL4, and VL3: *Pseudosydowia* sp. VL3.

### 3.5 Endophytic colonization of tomato plant by fungal isolates

Endophytic colonization was assessed by recovering the inoculated fungal isolates from the roots, stems, and leaves of tomato plants. After a two-week inoculation period, all 5 fungal isolates were successfully recovered from various tissues of host plants, indicating their systemic colonization ability in tomato plant.

### 3.6 Molecular identification of antagonistic endophytes

Five endophytic isolates including *Trichoderma* sp. KL1, *Trichoderma* sp. 8S1, *Chaetomium* sp. DL4, *Phaeophleospora* sp. XL4, and *Pseudosydowia* sp. VL3 with antagonistic effect on pathogens, were identified based on the morphological features and molecular data.

**Figure 8:**
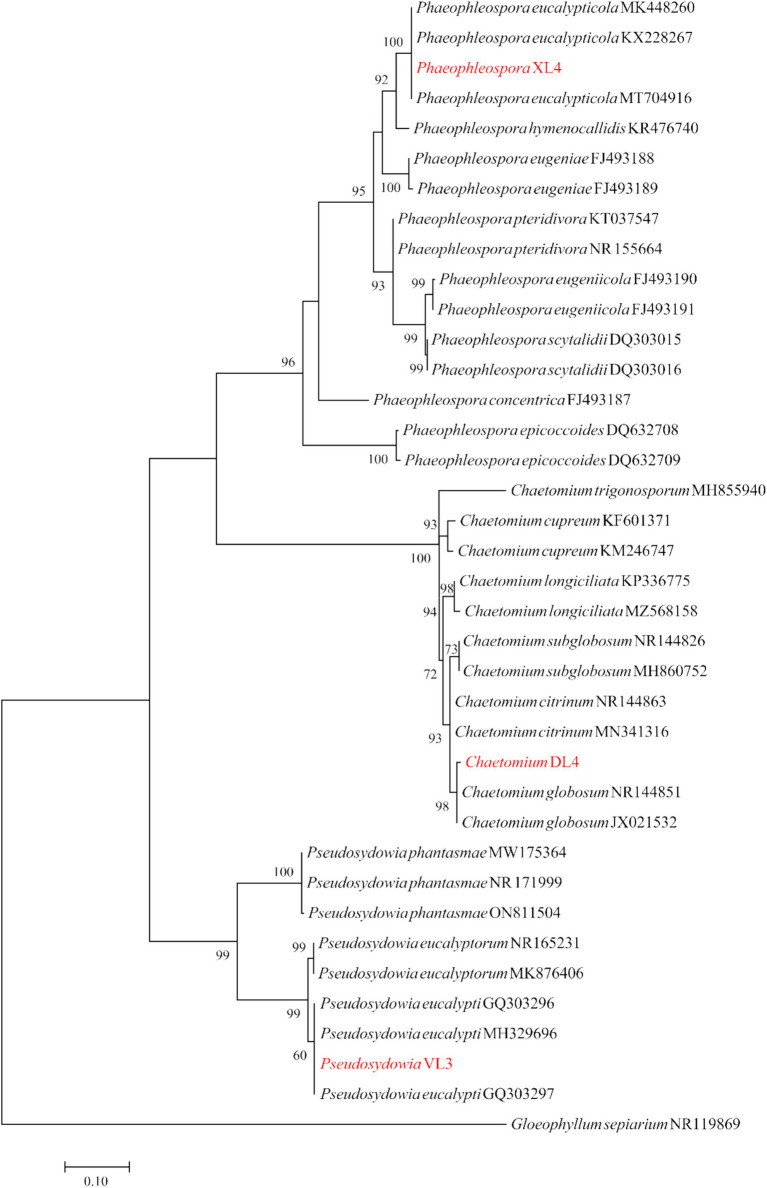
Maximum Likelihood (ML) tree based on aligned sequences of ITS gene of 35 isolates generated in MEGA 10 under K2 + G + I model. The tree was rooted to *Gloeophyllum sepiarium*. Bootstrap values (1,000 replicates) indicated at the nodes. The scale bar indicates nucleotide substitution in ML analysis, values ≥50% are shown above/below the branches. The surveyed isolates in the current study are highlighted in red.

**Figure 9:**
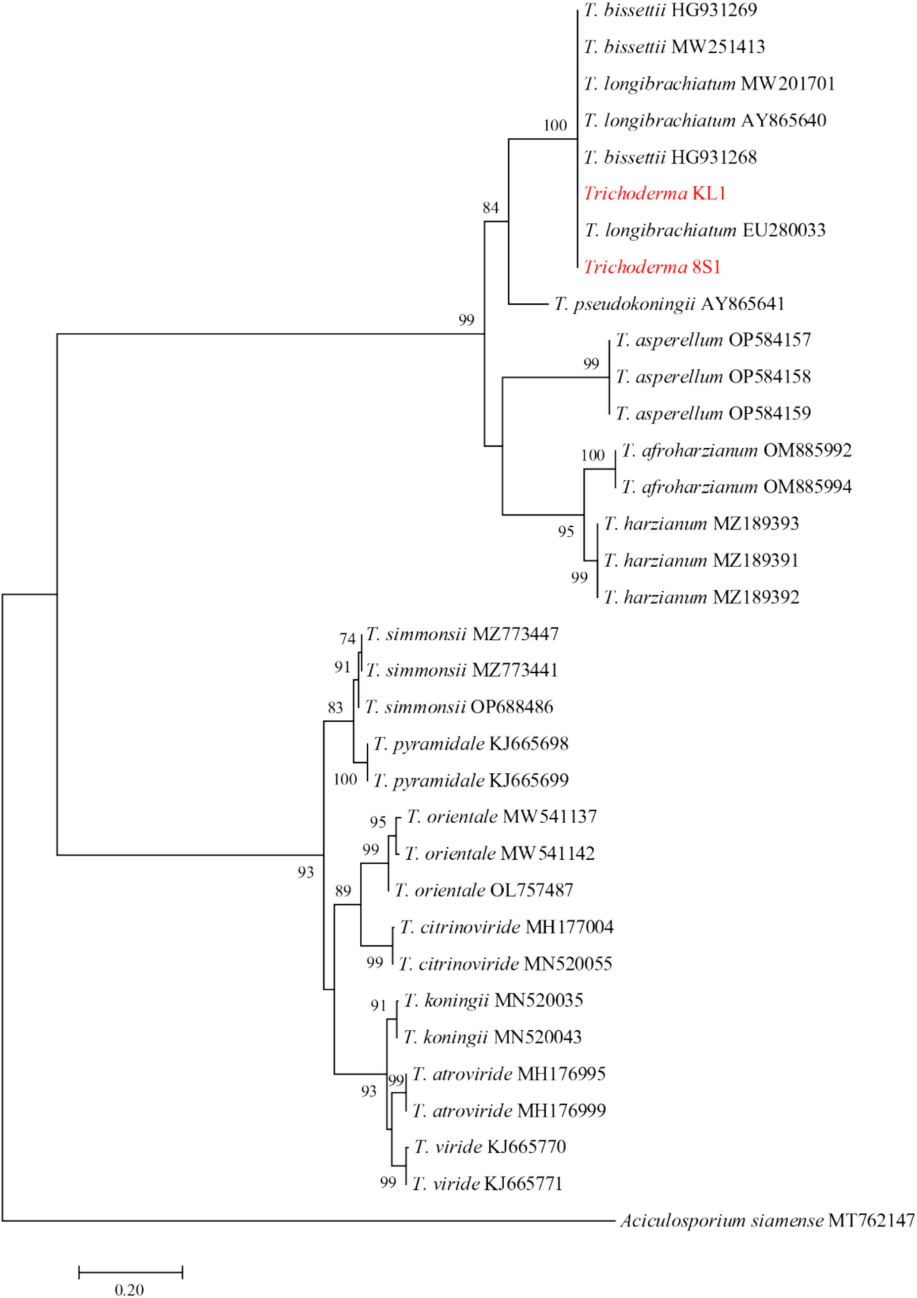
Maximum Likelihood (ML) tree based on aligned sequences of *tef-1α* gene of 31 isolates generated in MEGA 10 under HKY + G model. The tree was rooted to *Aciculosporium siamense*. Bootstrap values (1,000 replicates) indicated at the nodes. The scale bar indicates nucleotide substitution in ML analysis, values ≥50% are shown above/below the branches. The surveyed isolates in the current study are highlighted in red.

The ITS region was sequenced for isolates *Chaetomium* sp. DL4, *Phaeophleospora* sp. XL4, and *Pseudosydowia* sp. VL3, while the *ef-1α* partial gene was sequenced for *Trichoderma* isolates. Analysis revealed that isolates DL4, VL3, and XL4 clustered separately, corresponding to *Chaetomium globosum* Kunze, *Pseudosydowia eucalypti* (Verwoerd and du Plessis) Thambug. and K.D. Hyde, and *Phaeophleospora eucalypticola* Crous and M.J. Wingf. species, respectively (Figure 8). Furthermore, isolates 8S1 and KL1 were grouped with *Trichoderma longibrachiatum* Rifai and *Trichoderma bissettii* Sand.-Den. and Guarro (Figure 9). Since these species are inseparable via the *ef-1α* region, but differ morphologically, isolates KL1 and 8S1 were confirmed as *T. longibrachiatum* through a combination of morphological analysis and molecular data comparison.

## 4 Discussion

Endophytic fungi can enhance plant defense, degrade fungal structures, or provide essential nutrients, thereby significantly strengthening their antagonistic capabilities. Chitinases, enzymes that break down insoluble polymers, play a vital role in various fungal processes, including hyphal tube growth, sporulation, spore germination, cell division, and mycoparasitism against other microbes (De Marco et al., 2000; Karlsson and Stenlid, 2008). Plants utilize chitinases as a defensive mechanism against pathogens (Regalado et al., 2000; Hietala et al., 2004; Onaga and Taira, 2008) Furthermore, cellulases produced by endophytic fungi can contribute to plant defense by stimulating their immune responses (Amirita et al., 2012).

The aim of this study was isolation, identification, and selection of endophytic isolates with biocontrol potential against some important pathogens. Therefore, various tests were conducted *in vitro* and in greenhouse conditions. Among 754 fungal isolates obtained from 44 plant samples, 27 fungal genera were identified. Overall, the most prevalent endophytic isolates were found within the *Neofusicoccum*, *Cladosporium*, *Didymosphaeria*, and *Chaetomium* genera. In the study conducted by Fisher et al. (1993) on endophytic fungi found in *Eucalyptus* leaves and branches, the most common species identified were *Botryosphaeria dothidea* (Moug.) Ces. and De Not., and *Cytospora eucalypticola* Van der Westh. Also, Lupo et al. (2001), focusing on isolating species from flowers, capsules, and seeds of *E. globulus* Labill., identified *Cytospora chrysosperma* (Pers.) Fr., *Fusicoccum eucalypti* Sousa da Câmara, *Alternaria alternata* (Fr.) Keissl., *Fairmaniella leprosa* (Fairm.) Petr. and Syd., *Aureobasidium pullulans* (de Bary and Löwenthal) G. Arnaud, and *Cladosporium cladosporioides* (Fresen.) G.A. de Vries as endophytes. Further research by Lacerda et al. (2018) into the fungal endophytes’ communities of *E. microcorys* led to the discovery of *Castanediella eucalypticola* Crous and M.J. Wingf., and *Neophaeomoniella eucalypti* Roon.-Lath. and Crous in Brazil. An investigation into the endophytes of *E. globulus* twigs yielded 127 fungal isolates, including *Pringsheimia smilacis* E. Müll., *Lophiostoma corticola* (Fuckel) E.C.Y. Liew, Aptroot and K.D. Hyde, *Hormonema* sp., *Neofusicoccum luteum* (Pennycook and Samuels) Crous, Slippers and A.J.L. Phillips, *Phaeomoniella effuse* Damm and Crous, and *Ulocladium* sp., all identified as laccase positive strains (Fillat et al., 2016).

When different microbial species coexist within the same plant, endophytes and the host plant secrete metabolites that inhibit the growth of harmful microorganisms (Latz et al., 2018). Some endophytic fungi can suppress plant pathogens through various mechanisms, including induced resistance, antibiosis, hyperparasitism, and competition (Latz et al., 2018; Wei et al., 2019). These mechanisms may vary depending on the specific pathogen and can be employed individually or in combination (Wei et al., 2019; Aleahmad and Ebrahimi, 2023). Hyperparasitism is a mechanism employed by endophytes to protect their host plants against pathogens. In this process, endophytes directly attack pathogens by twisting and penetrating their hyphae and destroying their cell walls through the production of lyases (Fadiji and Babalola, 2020). Endophytes have been found to contain antibiotics and secondary metabolites with antimicrobial properties, including flavonoids, peptides, quinones, alkaloids, phenols, phenolic acids, steroids, terpenoids, VOCs, benzopyranones, chinones, saponins, tannins, tetralones, and xanthones, polyketides and different enzymes (Lugtenberg et al., 2016; Strobel, 2018; Elawady et al., 2023). VOCs are organic chemicals that readily evaporate at room temperature and pressure. Characterized by low molecular weights and high vapor pressures, they are easily transported in air and soil after release (Poveda, 2021). Over 250 different VOCs, primarily acids, alcohols, aldehydes, aromatics, esters, heterocycles, ketones, terpenes, and thiols, have been identified in fungi. These VOCs can be broadly classified into five categories: terpenoids, fatty acid derivatives, benzene compounds, acetone, and amino acid derivatives (Ling et al., 2024). In the current research, antifungal properties of purified endophytic fungi were evaluated against the pathogenic fungi *B. cinerea*, *F. oxysporum* f. sp. *lycopersici*, *M. phaseolina*, and *R. solani* through dual culture, VOC production, and enzyme production tests. Findings revealed that all tested endophytes exhibited over 85% inhibitory activity against *B. cinerea* in both dual culture and VOC tests. Furthermore, they significantly hindered the growth of *M. phaseolina* mycelia by up to 63%, and reduced microsclerotia formation. Against *F. oxysporum* f. sp. *lycopersici*, all isolates demonstrated over 68% inhibitory activity in dual culture test, although *Ch. Globosum* DL4 showed variable results in VOC production. Moreover, most isolates inhibited *R. solani* growth by approximately 70% in dual culture test, with *T. longibrachiatum* KL1 also displaying inhibitory activity in VOC production. Previous studies have demonstrated the antifungal potential of endophytes, particularly *T. longibrachiatum* and *T. harzianum* Rifai. Rajani et al. (2021) reported over 88% inhibition of mycelial growth for pathogens including *S. sclerotiorum* (Lib.) de Bary, *S. rolfsii* Sacc. 1911, *M. phaseolina*, and *F. oxysporum* in dual culture test. Additionally, VOC production test indicated inhibitory activity exceeding 50% against *S. sclerotiorum*, *Sclerotium rolfsii*, and *F. oxysporum*, but not against *M. phaseolina*.

Another study highlighted the antifungal potential of endophyte strains isolated from rice leaves, including *Paecilomyces tenuis* Y.F. Han and Z.Q. Liang, *Talaromyces pinophilus* (Hedgc.) Samson, N. Yilmaz, Frisvad and Seifert, *Nigrospora sphaerica* (Sacc.) E.W. Mason, *Nigrospora oryzae* (Berk. and Broome) Petch, *Aspergillus terreus* Thom, and *T. longibrachiatum*. These strains demonstrated inhibitory activity against pathogens like *Magnaporthe grisea* (T.T. Hebert) M.E. Barr, *M. phaseolina*, *Pythium* sp., *F. oxysporum*, *R. solani*, and *Colletotrichum falcatum* Went in dual culture test. Notably, *T. longibrachiatum* also showed inhibitory activity in VOC production test (Roy and Banerjee, 2019).

Endophytes with the capacity to secrete extracellular chitinase contribute to the degradation of chitin, a *β*-(1,4)-linked polymer of N-acetyl-D-glucosamine, and the cell wall structure of most phytopathogenic fungi, alongside the synthesis of other multifaceted bioactive compounds (Hartl et al., 2012; Ebrahimi et al., 2022). Endophytes, including non-pathogenic microorganisms, can induce systemic resistance and activate specific genes involved in pathogenesis (Fadiji and Babalola, 2020). Some endophytes can degrade plant lignin and cellulose and secrete chitinase, which induces the host plant’s immune system and decomposes the cell walls of phytopathogenic fungi, respectively (Ebrahimi et al., 2022). The current study revealed that all isolates, except *Ps. eucalypti* VL3, could produce cellulase, and all isolates produced chitinase, with *Trichoderma* sp. 8S1 exhibiting the highest levels of both. Pedrero-Méndez et al. (2021) reported significant cellulolytic and chitinolytic activity in various *Trichoderma* sp. endophytes isolated from wheat. Additionally, a *Trichoderma* sp. from almonds was found to secrete chitinase, which degraded the cell wall of *B. cinerea* and mitigated disease symptoms (Aoki et al., 2020). Endophytic *Trichoderma viride* Pers. and *Trichoderma koningii* Oudem., through chitinase, protease, and glucanase production, degraded *M. phaseolina* cell wall, significantly inhibiting its growth (Gajera et al., 2023). Research by Ebrahimi et al. (2022) highlighted cellulolytic activity in apple-isolated endophytes like *Aureobasidium microstictum* (Bubák) W.B. Cooke, *Fusarium lateritium* Nees 6, and *Coniochaeta endophytica* A.H. Harrington and A.E. Arnold, with *Ch. Globosum* and *F. lateritium* displaying chitinolytic activity. Further studies on endophytes from medicinal plants like *Terminalia catappa* and *T. mantaly* revealed high cellulase production by *Penicillium chermesinum* Biourge (Toghueo et al., 2017).

Endophytic microorganisms, similar to rhizosphere microbes, play a vital role in plant growth and development (Santoyo et al., 2016). These fungi can enhance the fitness and growth of host plants by facilitating the production of phytohormones, siderophores, indolic compounds, phosphate solubilization and nutrients production such as polysaccharides, lipids, minerals and vitamins (Sharma and Goyal, 2017; Ling et al., 2024). The solubilization of inorganic insoluble phosphate (Plants can then absorb this soluble phosphate) by various microorganisms depends on their ability to produce organic acids in their specific environment (Sharma and Goyal, 2017). This study showed the phosphate solubilization capabilities of the endophytic fungi *T. longibrachiatum* KL1, *T. longibrachiatum* 8S1, and *Ch. Globosum* DL4. Previous studies have also identified phosphate-solubilizing endophytes, such as *T. longibrachiatum* isolated from peanuts (Al-Askar et al., 2022), *C. endophytica* and *F. lateritium* (Ebrahimi et al., 2022), and various species associated with cucumbers, including *Aspergillus niger* Tiegh., *Aspergillus japonicus* Saito, *T. viride*, and *Ch. Globosum* (Yadav et al., 2020). These endophytes can contribute to plant growth promotion by solubilizing phosphate.

The fungus *Ps. eucalypti*, previously known as *Sphaerulina eucalypti* Verwoerd and du Plessis, was identified in 1931 as the causative agent of leaf spot disease in African *Eucalyptus* trees (Verwoerd, 1931). Recent studies in California highlighted its frequent association with *Eucalyptus* decline and dieback, characterized by leaf spots and shoot tip necrosis. The presence of *Ps. eucalypti* in *Eucalyptus* cultivation areas suggests its potential endophytic lifestyle within the plant (Garbelotto et al., 2021). Additionally, *Ph. eucalypticola*, reported in Australia (Crous et al., 2016), was later identified as a pathogen on pine species in South Korea (Choi et al., 2022). This study presents the first documented occurrence of *Ps. eucalypti* and *Ph. eucalypticola* isolates in Iran, establishing their global presence as endophytes. Research highlights the variability in pathogenicity among fungal isolates, with some being non-pathogenic and capable of suppressing pathogenic species when applied preemptively to host plants (Alabouvette et al., 2009; Kaur et al., 2011; Iida et al., 2022; Ebrahimi et al., 2023).

Such interactions can either directly or indirectly mitigate disease progression, making non-pathogenic strains valuable biological control agents (Alabouvette et al., 1993; Kaur et al., 2011; Saito et al., 2021; Ebrahimi et al., 2023). These strains can induce systemic resistance within plants, as demonstrated by the use of non-pathogenic *F. oxysporum* strains to manage Fusarium wilt (Iida et al., 2022) and non-pathogenic *F. fujikuroi* strains to control *B. cinerea* and *M. phaseolina* (Ebrahimi et al., 2023). Furthermore, non-pathogenic *Fusarium* spp. strains effectively suppressed rice bakanae disease (Saito et al., 2021), underscoring the potential of non-pathogenic fungal strains in crop protection strategies. Greenhouse tests evaluated the efficacy of selected fungal isolates as biological control agents against specific plant diseases. Among the investigated isolates, *Ph. eucalypticola* XL4 controlled gray mold disease by 63%, while *T. longibrachiatum* KL1 achieved complete control (100%), and *T. longibrachiatum* 8S1 reached 98%. However, *Ph. eucalypticola* XL4 was less effective against Fusarium wilt disease, controlling it by only 37%, compared to other isolates that exceeded 75% control. For charcoal rot disease, *T. longibrachiatum* 8S1 and *Ch. Globosum* DL4 surpassed 70% control, while others reached over 80%. In combating Rhizoctonia damping-off disease, *T. longibrachiatum* isolates consistently demonstrated superior control, exceeding 80%, followed by other isolates with varying degrees of success. Studies by Tadayyon Rad and Ebrahimi (2023), showed that *Ch. globosum* isolates inhibited *M. phaseolina* mycelial growth by over 67% *in vitro* and 90% in greenhouse. Park et al. (2019) highlighted *Trichoderma* sp. potential in controlling grey mold and charcoal rot diseases through mechanisms like phosphate solubilization, chitinase production, and cellulase activity, significantly reducing disease symptoms in tomato plant. Endophytic *Paecilomyces formosus* Sakag., May. Inoue and Tada ex Houbraken and Samson, isolated from tomato plants, demonstrated the ability to degrade *R. solani* cell walls using various enzymes, alongside phosphate and zinc solubilization, leading to a significant reduction in disease symptoms in greenhouse conditions. Further research involving *Rhizoctonia* and *Fomes* isolates from wheat and wild barley demonstrated their potential to reduce symptoms caused by *F. oxysporum* f. sp. *lycopersici* and inhibit mycelial growth. Among these isolates, *Coprinopsis urticicola* (Berk. and Broome) Redhead, Vilgalys and Moncalvo was the only one found to be effective in greenhouse.

The evidences indicate that the effectiveness of antagonist factors, including endophytes, depends on the pathogen (even different isolates of the same pathogen) and the host plant (Ebrahimi et al., 2023). The interactions between plants and endophytes are very complex and vary from host to host, and endophyte to endophyte (Gupta et al., 2020; Ebrahimi et al., 2023). Endophytic fungal isolates demonstrated significant inhibitory effects against *B. cinerea*. Among the isolates tested, *T. longibrachiatum* KL1 and *T. longibrachiatum* 8S1 exhibited the highest inhibition rates, reaching 100 and 98%, respectively. In contrast, *Ph. eucalypticola* XL4 showed the lowest inhibition, with approximately 63%. Similarly, endophytic fungal isolates demonstrated varying levels of inhibition against *F. oxysporum* f. sp. *lycopersici*. *T. longibrachiatum* KL1 exhibited the highest inhibitory effect, reaching approximately 92%, while *Ph. eucalypticola* XL4 showed the lowest inhibition at around 37%. Additionally, *T. longibrachiatum* KL1 exhibited the highest inhibition rate of approximately 87%, while *T. longibrachiatum* 8S1 also showed a considerable inhibitory effect of around 70% against charcoal rot agent. Endophytic fungi, particularly *T. longibrachiatum* KL1 and 8S1, significantly reduced the incidence of Rhizoctonia disease caused by *R. solani*. These *Trichoderma* isolates exhibited the highest inhibition rates, exceeding 80%, while *Ps. eucalypti* VL3 showed a lower inhibition rate of 64%. These results confirmthat the effectiveness of endophytic antagonism depends on pathogen and host plant. Studies conducted by Ebrahimi et al. (2023) showed that endophyte isolates *Ch. globosum* 2S1, *Ch. globosum* 3 L2, *F*. *acuminatum* Ellis and Everh., *F. fujikuroi*, and *F. incarnatum* (Desm.) Sacc. exhibited different levels of inhibitory effect against the pathogens *B. cinerea* and *M. phaseolina*.

## 5 Conclusion

Endophytic fungi are recognized as a vast source with strong potential in biocontrol biotic stresses in agriculture. This study has identified *T. longibrachiatum* KL1 and *T. longibrachiatum* 8S1 as promising endophytic fungal isolates capable of inhibiting gray mold, Fusarium wilt, charcoal disease, and Rhizoctonia damping-off in tomato plants under greenhouse conditions. Results suggest that the reduction of disease caused by the mentioned pathogens is attributed to various mechanisms, including the production of volatile and permeable compounds in the culture medium and the secretion of enzymes such as cellulase and chitinase by endophytic fungi. These isolates demonstrated promising compounds derived from endophytes, which could contribute to developing more sustainable approaches for managing plant diseases. Further research is necessary to elucidate the fundamental mechanisms underlying the interactions between endophytic fungi and their host plants. Additionally, it is crucial to validate these findings under field conditions and across a wider range of host plants to ensure the functionality and effectiveness of these biocontrol agents in diverse agricultural ecosystems.

## Data Availability

The datasets presented in this study can be found in online repositories. The names of the repository/repositories and accession number(s) can be found in the article/Supplementary material.
