# Supplementary material for: Antagonism of Eucalyptus endophytic fungi against some important crop fungal diseases
Source: Front Microbiol. 2025 Feb 12;16:1523127. doi: 10.3389/fmicb.2025.1523127 (PMC11861536; doi:10.3389/fmicb.2025.1523127)
Supplement: Supplementary file 1 [file Table_1.DOCX]

Supplementary Material

**Supplementary Table**

**Supplementary Table S1** Details of sequences obtained from the Genbank for use in phylogenetic analyses.

| **Genbank accession number** | **Geographical region** | **Host** | **Collection accession number** | **Species** |
| --- | --- | --- | --- | --- |
| ***Tef-1α*** |  |  |  |  |
|  | **Iran** | ***Eucalyptus camaldulensis*** |  | ***Trichoderma longibrachiatum*** |
| HG931269 | Spain | Human bone | UTHSC:12-337 | *Trichoderma bissettii* |
| MW251413 | South Korea | marine sponge | GJ-Sp1 | *Trichoderma bissettii* |
| MW201701 | Italy | compost | Tl35 | *Trichoderma longibrachiatum* |
| AY865640 | Austria | *-* | CBS 816.68 | *Trichoderma longibrachiatum* |
| HG931268 | USA | Human foot | UTHSC:11-455 | *Trichoderma bissettii* |
| EU280033 | Colombia | *-* | HBSY3 | *Trichoderma longibrachiatum* |
| AY865641 | Austria | *-* | DAOM 167678 | *Trichoderma pseudokoningii* |
| OP584157 | South Korea | sea sand | SFC20220920-B481 | *Trichoderma asperellum* |
| OP584158 | South Korea | sea sand | SFC20220920-B491 | *Trichoderma asperellum* |
| OP584159 | South Korea | sea sand | SFC20220920-B484 | *Trichoderma asperellum* |
| OM885992 | Brazil | *Cymbopogon winterianus* | NPF Tricho Bsb 3 | *Trichoderma afroharzianum* |
| OM885994 | Brazil | *Cymbopogon winterianus* | NPF Tricho Bsb 5 | *Trichoderma afroharzianum* |
| MZ189393 | Canada | *Vitis vinifera* | SuRDC-1448 | *Trichoderma harzianum* |
| MZ189391 | Canada | *Vitis vinifera* | SuRDC-1441 | *Trichoderma harzianum* |
| MZ189392 | Canada | *Vitis vinifera* | SuRDC-1447 | *Trichoderma harzianum* |
| MZ773447 | Hungary | *Lentinula edodes* cultivation | SZMC 24248 | *Trichoderma simmonsii* |
| MZ773441 | Serbia | *Lentinula edodes* cultivation | SZMC 25740 | *Trichoderma simmonsii* |
| OP688486 | Netherlands | *-* | EEK_C4 | *Trichoderma simmonsii* |
| KJ665698 | *Italy* | *-* | S573 | *Trichoderma pyramidale* |
| KJ665699 | Italy | *-* | CBS:135574 | *Trichoderma pyramidale* |
| MW541137 | Peru | soil of cacao farm | CP01_3 | *Trichoderma orientale* |
| MW541142 | Peru | soil of cacao farm | CP63_1 | *Trichoderma orientale* |
| OL757487 | Italy | *-* | F6 | *Trichoderma orientale* |
| MH177004 | Hungary | settled dust, office, Espoo | SJ40 | *Trichoderma citrinoviride* |
| MN520055 | Hungary | soil | SZMC:26776 | *Trichoderma citrinoviride* |
| MN520035 | Austria | soil | SZMC:24287 | *Trichoderma koningii* |
| MN520043 | Austria | soil | SZMC:24290 | *Trichoderma koningii* |
| MH176995 | Helsinki | Andersen impactor, office | H3/226 | *Trichoderma atroviride* |
| MH176999 | Lahti | fallout plate, school | KIV10 | *Trichoderma atroviride* |
| KJ665770 | Spain | *-* | S472 | *Trichoderma viride* |
| KJ665771 | Spain | *-* | S552 | *Trichoderma viride* |
| MT762147 | Thailand | plant | BCC 85382 | *Aciculosporium siamense* |
| **ITS** |  |  |  |  |
| **PP320330** | **Iran** | ***Eucalyptus camaldulensis*** | **ABRIICC 10384** | ***Phaeophleospora eucalypticola*** |
| MK448260 | Taiwan | - | NTOU:4394 | *Phaeophleospora eucalypticola* |
| KX228267 | Netherlands | leaves of *Eucalyptus robusta* | CPC 26523 | *Phaeophleospora eucalypticola* |
| MT704916 | USA | - | FKII_L3_CM_PAB3 | *Phaeophleospora eucalypticola* |
| KR476740 | Thailand | leaves of epiphyte | CPC 25018 | *Phaeophleospora hymenocallidis* |
| FJ493188 | Brazil | *Eugenia* *uniflora* | CPC 15143 | *Phaeophleospora eugeniae* |
| FJ493189 | Brazil | *Eugenia* *uniflora* | CPC 15159 | *Phaeophleospora eugeniae* |
| KT037547 | Brazil | *Serpocaulon triseriale* | COAD:1182 | *Phaeophleospora pteridivora* |
| NR_155664 | Brazil | *Serpocaulon triseriale* | CPC:24683 | *Phaeophleospora pteridivora* |
| FJ493190 | Netherlands | - | CPC 2557 | *Phaeophleospora eugeniicola* |
| FJ493191 | Netherlands | - | CPC 2558 | *Phaeophleospora eugeniicola* |
| DQ303015 | Colombia | *Eucalyptus* sp. | CPC 10988 | *Phaeophleospora scytalidii* |
| DQ303016 | Colombia | *Eucalyptus* sp. | CPC 10998 | *Phaeophleospora scytalidii* |
| FJ493187 | Kenya | - | CPC 3615 | *Phaeophleospora concentrica* |
| DQ632708 | Indonesia | *Eucalyptus grandis* | MUCC430 | *Phaeophleospora epicoccoides* |
| DQ632709 | Indonesia | *Eucalyptus grandis* | CMW22483 | *Phaeophleospora epicoccoide* |
| MH855940 | Canada | - | CBS 146.38 | *Chaetomium trigonosporum* |
| KF601371 | Brazil | peanut seed | CH022 | *Chaetomium cupreum* |
| KM246747 | Malaysia | - | RHb 18S | *Chaetomium cupreum* |
| KP336775 | China | soil | LC4682 | *Chaetomium longiciliata* |
| MZ568158 | Israel | Mediterranean Sea | FS-22-1 | *Chaetomium longiciliata* |
| NR_144826 | - | - | MUCL 18694 | *Chaetomium subglobosum* |
| MH860752 | Turkey | - | CBS:483.73 | *Chaetomium subglobosum* |
| NR_144863 | - | - | CBS 693.82 | *Chaetomium citrinum* |
| MN341316 | South Korea | *Myelochroa entotheiochroa* | KoRLI046138 | *Chaetomium citrinum* |
|  | **Iran** | ***Eucalyptus camaldulensis*** |  | ***Chaetomium globosum*** |
| NR_144851 | - | - | CBS 160.62 | *Chaetomium globosum* |
| JX021532 | India | soil | TNAU Cg35 | *Chaetomium globosum* |
| MW175364 | Namibia | *Moringa* *ovalifolia* | CPC 38883 | *Pseudosydowia phantasmae* |
| NR_171999 | Namibia | *Moringa* *ovalifolia* | CBS 146830 | *Pseudosydowia phantasmae* |
| ON811504 | Namibia | *Moringa* *ovalifolia* | CBS:146982 | *Pseudosydowia phantasmae* |
| NR_165231 | Australia | *Eucalyptus* sp. | CBS 145546 | *Pseudosydowia eucalyptorum* |
| MK876406 | Australia | *Eucalyptus* sp. | CBS:145546 | *Pseudosydowia eucalyptorum* |
| **PP320331** | **Iran** | ***Eucalyptus camaldulensis*** | **ABRIICC 10385** | ***Pseudosydowia eucalypti*** |
| GQ303296 | Australia | *Eucalyptus* sp. | CPC:14028 | *Pseudosydowia eucalypti* |
| MH329696 | Brazil | *Eucalyptus microcorys* | LTL117 | *Pseudosydowia eucalypti* |
| GQ303297 | Portugal | *Eucalyptus* sp*.* | CPC:14927 | *Pseudosydowia eucalypti* |
| NR_119869 | USA | - | Wilcox-3BB | *Gloeophyllum sepiarium* |

The surveyed isolates in the current study are highlighted in bold.

**Supplementary Table S2** Antifungal activity of eucalyptus endophytic fungi in vitro.

| NO | Isolates | *B. cinerea* | *F. oxysporum* | *R. solani* | *M. phaseolina* |
| --- | --- | --- | --- | --- | --- |
| 1 | *Alternaria* sp. 13F1 | **-** | **-** | **-** | **-** |
| 2 | *Alternaria* sp. 14S1 | **-** | **-** | **-** | **-** |
| 3 | *Alternaria* sp. 14S3 | **-** | **-** | **-** | **-** |
| 4 | *Alternaria* sp. DL2 | **-** | **+** | **-** | **+** |
| 5 | *Alternaria* sp. GS1 | **++** | **++** | **+** | **+** |
| 6 | *Alternaria* sp. IL2 | **-** | **-** | **-** | **-** |
| 7 | *Alternaria* sp. JL2 | **-** | **+** | **-** | **-** |
| 8 | *Alternaria* sp. RF2 | **-** | **-** | **-** | **-** |
| 9 | *Alternaria* sp. RL1 | **-** | **-** | **-** | **-** |
| 10 | *Alternaria* sp. TL2 | **-** | **-** | **-** | **-** |
| 11 | *Alternaria* sp. UF1 | **-** | **+** | **-** | **+** |
| 12 | *Alternaria* sp. VF1B | **+** | **+** | **-** | **+** |
| 13 | *Alternaria* sp. VL4 | **-** | **-** | **-** | **-** |
| 14 | *Alternaria* sp. VL6B | **-** | **-** | **-** | **-** |
| 15 | *Alternaria* sp. WF1 | **-** | **-** | **-** | **-** |
| 16 | *Alternaria* sp. XF1 | **-** | **-** | **-** | **-** |
| 17 | *Alternaria* sp. YL2 | **-** | **-** | **-** | **-** |
| 18 | *Alternaria* sp. YS1 | **-** | **-** | **-** | **-** |
| 19 | *Aspergillus* sp. 15F1 | **-** | **-** | **+** | **-** |
| 20 | *Aspergillus* sp. 15L1 | **-** | **-** | **-** | **-** |
| 21 | *Aspergillus* sp. 17L2 | **+** | **-** | **-** | **-** |
| 22 | *Aspergillus* sp. 18L1 | **+** | **++** | **+** | **++** |
| 23 | *Aspergillus* sp. 8L1 | **-** | **-** | **-** | **-** |
| 24 | *Aspergillus* sp. 9L1B | **+** | **++** | **+** | **++** |
| 25 | *Aspergillus* sp. BL2A | **+** | **++** | **+** | **++** |
| 26 | *Aspergillus* sp. BL2B | **++** | **++** | **++** | **++** |
| 27 | *Aspergillus* sp. ES1 | **-** | **-** | **-** | **-** |
| 28 | *Aspergillus* sp. GL1 | **+** | **++** | **+** | **+** |
| 29 | *Aspergillus* sp. KS2 | **+** | **++** | **++** | **++** |
| 30 | *Aspergillus* sp. NS1 | **-** | **-** | **-** | **+** |
| 31 | *Aspergillus* sp. VL1C | **-** | **-** | **-** | **-** |
| 32 | *Aspergillus* sp. XF3 | **-** | **-** | **-** | **-** |
| 33 | *Bipolaris* sp. OL1 | **-** | **-** | **-** | **-** |
| 34 | *Bipolaris* sp. OL2 | **+** | **+** | **+** | **+** |
| 35 | *Bipolaris* sp. YL1 | **-** | **-** | **-** | **-** |
| 36 | *Bipolaris* sp. YL3 | **-** | **+** | **-** | **+** |
| 37 | *Botrytis* sp. HS1 | **-** | **++** | **-** | **+** |
| 38 | *Chaetomium* sp. 17L1 | **++** | **-** | **-** | **-** |
| 39 | *Chaetomium* sp. 17L3 | **++** | **++** | **-** | **+** |
| 40 | *Chaetomium* sp. 2L4 | **+** | **+** | **-** | **+** |
| 41 | *Chaetomium* sp. 3L2 | **+** | **-** | **+** | **+** |
| 42 | *Chaetomium* sp. 6L2 | **-** | **-** | **-** | **-** |
| 43 | *Chaetomium* sp. 7L31 | **-** | **-** | **-** | **-** |
| 44 | *Chaetomium* sp. CHA-VF1 | **-** | **-** | **-** | **-** |
| 45 | *Chaetomium* sp. CHA-WS1 | **++** | **++** | **-** | **++** |
| 46 | *Chaetomium* sp. DL1 | **-** | **++** | **-** | **-** |
| 47 | *Chaetomium* sp. DL3 | **++** | **++** | **-** | **+** |
| 48 | *Chaetomium* sp. DL4 | **++** | **+** | **+** | **+** |
| 49 | *Chaetomium* sp. KF2 | **+** | **+** | **-** | **+** |
| 50 | *Chaetomium* sp. KS1A | **++** | **+** | **-** | **++** |
| 51 | *Chaetomium* sp. ML1 | **-** | **-** | **-** | **-** |
| 52 | *Chaetomium* sp. ML4 | **+** | **+** | **-** | **+** |
| 53 | *Cladosporium* sp. 10L2A | **-** | **-** | **-** | **-** |
| 54 | *Cladosporium* sp. 18S2 | **-** | **-** | **-** | **-** |
| 55 | *Cladosporium* sp. 7L1 | **-** | **-** | **+** | **-** |
| 56 | *Cladosporium* sp. 7L2 | **+** | **+** | **-** | **-** |
| 57 | *Cladosporium* sp. 8L2 | **-** | **-** | **-** | **-** |
| 58 | *Cladosporium* sp. 9L1A | **-** | **-** | **-** | **-** |
| 59 | *Cladosporium* sp. 9L2 | **-** | **-** | **-** | **-** |
| 60 | *Cladosporium* sp. AL1 | **-** | **-** | **-** | **-** |
| 61 | *Cladosporium* sp. FL1 | **-** | **-** | **-** | **-** |
| 62 | *Cladosporium* sp. GL3 | **-** | **-** | **-** | **-** |
| 63 | *Cladosporium* sp. HL1A | **-** | **-** | **-** | **-** |
| 64 | *Cladosporium* sp. HL3 | **-** | **-** | **-** | **-** |
| 65 | *Cladosporium* sp. IL1 | **-** | **-** | **+** | **-** |
| 66 | *Cladosporium* sp. IL4 | **-** | **-** | **-** | **-** |
| 67 | *Cladosporium* sp. JL1 | **-** | **-** | **-** | **-** |
| 68 | *Cladosporium* sp. OS1 | **++** | **-** | **-** | **-** |
| 69 | *Cladosporium* sp. SS3A | **-** | **-** | **-** | **-** |
| 70 | *Cladosporium* sp. VF1A | **-** | **-** | **-** | **-** |
| 71 | *Cladosporium* sp. VF2A | **-** | **-** | **-** | **-** |
| 72 | *Cladosporium* sp. VL1A | **++** | **++** | **+** | **++** |
| 73 | *Cladosporium* sp. VL2A | **-** | **-** | **-** | **-** |
| 74 | *Cladosporium* sp. VS1A | **-** | **-** | **+** | **+** |
| 75 | *Cladosporium* sp. VS2A | **-** | **-** | **-** | **-** |
| 76 | *Cladosporium* sp. XS2 | **-** | **-** | **-** | **-** |
| 77 | *Cladosporium* sp. XS4 | **-** | **-** | **-** | **-** |
| 78 | *Cladosporium* sp. ZL1 | **-** | **-** | **-** | **-** |
| 79 | *Cladosporium* sp. ZS1 | **-** | **-** | **-** | **-** |
| 80 | *Coniosporium* sp. SS2 | **-** | **-** | **-** | **-** |
| 81 | *Cytospora* sp. 15F3 | **-** | **-** | **-** | **-** |
| 82 | *Cytospora* sp. 5L2 | **-** | **++** | **-** | **+** |
| 83 | *Cytospora*sp. 15F2 | **-** | **-** | **-** | **-** |
| 84 | *Didymella* sp. LS1 | **+** | **+** | **-** | **+** |
| 85 | *Didymosphaeria* sp. !X! | **-** | **-** | **-** | **-** |
| 86 | *Didymosphaeria* sp. 16L1 | **-** | **-** | **-** | **-** |
| 87 | *Didymosphaeria* sp. HL1B | **+** | **+** | **+** | **+** |
| 88 | *Didymosphaeria* sp. IL3 | **-** | **-** | **-** | **-** |
| 89 | *Didymosphaeria* sp. JL3 | **-** | **-** | **-** | **-** |
| 90 | *Didymosphaeria* sp. KF1 | **-** | **-** | **-** | **-** |
| 91 | *Didymosphaeria* sp. KL2 | **-** | **-** | **-** | **-** |
| 92 | *Didymosphaeria* sp. XF2 | **-** | **+** | **-** | **-** |
| 93 | *Didymosphaeria* sp. XL1 | **-** | **-** | **-** | **-** |
| 94 | *Didymosphaeria* sp. XS1 | **-** | **-** | **-** | **+** |
| 95 | *Fusarium* sp. VS1C | **-** | **-** | **-** | **-** |
| 96 | *Gymnoascus* sp. HL2 | **-** | **-** | **-** | **++** |
| 97 | *Valsonectria* sp. BL4 | **-** | **-** | **-** | **-** |
| 98 | Iodophanus sp. 13F2A | **+** | **+** | **-** | **+** |
| 99 | *Iodophanus* sp. 13F2B | **-** | **-** | **-** | **-** |
| 100 | *Iodophanus* sp. 13F3 | **-** | **+** | **-** | **-** |
| 101 | *Microsphaeropsis* sp. 14S2 | **-** | **-** | **-** | **-** |
| 102 | *Neofusicoccum* sp. ML2 | **-** | **+** | **-** | **-** |
| 103 | *Neofusicoccum* sp. MS1 | **-** | **-** | **-** | **+** |
| 104 | *Neofusicoccum* sp. RF1A | **-** | **+** | **+** | **-** |
| 105 | *Neofusicoccum* sp. RF1B | **-** | **-** | **-** | **-** |
| 106 | *Neofusicoccum* sp. RL2 | **-** | **-** | **-** | **-** |
| 107 | *Neofusicoccum* sp. SL1 | **+** | **++** | **+** | **+** |
| 108 | *Neofusicoccum* sp. TF1 | **++** | **++** | **++** | **++** |
| 109 | *Neofusicoccum* sp. TL1 | **-** | **-** | **-** | **-** |
| 110 | *Neofusicoccum* sp. TL3 | **-** | **-** | **-** | **-** |
| 111 | *Neofusicoccum* sp. TL4 | **-** | **+** | **-** | **-** |
| 112 | *Neofusicoccum* sp. TL5 | **-** | **-** | **-** | **-** |
| 113 | *Neofusicoccum* sp. TL6 | **++** | **++** | **++** | **++** |
| 114 | *Neofusicoccum* sp. UL1 | **-** | **-** | **-** | **-** |
| 115 | *Neofusicoccum* sp. UL3 | **-** | **-** | **-** | **-** |
| 116 | *Neofusicoccum* sp. US1 | **-** | **-** | **-** | **-** |
| 117 | *Neofusicoccum* sp. VF2B |  | **++** | **++** | **+** |
| 118 | *Neofusicoccum* sp. XF4 | **+** | **++** | **+** | **++** |
| 119 | *Schizothecium* sp. JF1 | **-** | **-** | **-** | **-** |
| 120 | *Schizothecium* sp. JF2 | **-** | **-** | **-** | **-** |
| 121 | *Niesslia* sp. EL1 | **-** | **-** | **-** | **-** |
| 122 | *Niesslia* sp. SS1 | **+** | **-** | **-** | **-** |
| 123 | *Niesslia* sp. SS3B | **-** | **-** | **-** | **-** |
| 124 | *Paecillomyces* sp. ML3 | **-** | **-** | **-** | **-** |
| 125 | *Penicillium* sp. 13L1 | **-** | **-** | **-** | **-** |
| 126 | *Penicillium* sp. 13S1 | **+** | **-** | **-** | **-** |
| 127 | *Penicillium* sp. 2S1 | **-** | **-** | **-** | **+** |
| 128 | *Penicillium* sp. 3L1 | **-** | **+** | **-** | **+** |
| 129 | *Penicillium* sp. 5L1 | **+** | **+** | **-** | **-** |
| 130 | *Penicillium* sp. 6L1B | **++** | **-** | **-** | **++** |
| 131 | *Penicillium* sp. BS1 | **-** | **++** | **-** | **+** |
| 132 | *Penicillium* sp. KS1B | **++** | **++** | **++** | **+** |
| 133 | *Penicillium* sp. PS1 | **-** | **+** | **-** | **+** |
| 134 | *Penicillium* sp. VL1B | **-** | **-** | **-** | **+** |
| 135 | *Penicillium* sp. VL2B | **-** | **-** | **-** | **-** |
| 136 | *Penicillium* sp. VL3B | **++** | **++** | **+** | **+** |
| 137 | *Penicillium* sp. VL6A | **-** | **-** | **-** | **-** |
| 138 | *Penicillium* sp. VS1B | **-** | **-** | **-** | **-** |
| 139 | *Penicillium* sp. VS3 | **-** | **-** | **-** | **-** |
| 140 | *Pestalotiopsis* sp. VS2 | **-** | **+** | **-** | **-** |
| 141 | *Peziza* sp. 2L2 | **+** | **+** | **-** | **+** |
| 142 | *peziza* sp. CS1 | **-** | **-** | **-** | **+** |
| 143 | *Peziza* sp. CS2 | **-** | **-** | **-** | **+** |
| 144 | *Peziza* sp. WS1 | **-** | **-** | **-** | **-** |
| 145 | *phaeophleospora* sp. XL2 | **-** | **-** | **-** | **-** |
| 146 | *phaeophleospora* sp. XL4 | **+** | **+** | **+** | **++** |
| 147 | *pseudosydowia* sp. VL3 | **+** | **+** | **+** | **++** |
| 148 | *Rhizopus* sp. 6L3 | **-** | **-** | **-** | **-** |
| 149 | *Trichoderma s*p. 8S1 | **++** | **++** | **++** | **++** |
| 150 | *Trichoderma* sp. KL1 | **++** | **++** | **++** | **++** |
| 151 | *Ulocladium* sp. BL1 | **-** | **-** | **-** | **+** |
| 152 | *Ulocladium* sp. BL3 | **-** | **-** | **-** | **++** |
| 153 | QL1 (mycelia sterilia) | **++** | **-** | **-** | **-** |
| 154 | QL2 (mycelia sterilia) | **++** | **+** | **+** | **+** |
| 155 | JL4 (mycelia sterilia) | **-** | **-** | **-** | **-** |
| 156 | JL7 (mycelia sterilia) | **-** | **-** | **-** | **-** |
| 157 | JS1 (mycelia sterilia) | **-** | **-** | **-** | **-** |
| 158 | 13L2 (mycelia sterilia) | **-** | **-** | **-** | **-** |
| 159 | 13L3 (mycelia sterilia) | **-** | **-** | **-** | **-** |
| 160 | 13S2 (mycelia sterilia) | **-** | **-** | **-** | **-** |
| 161 | 18F1 (mycelia sterilia) | **+** | **-** | **+** | **+** |
| 162 | 18F2 (mycelia sterilia) | **-** | **-** | **-** | **-** |
| 163 | 18S1 (mycelia sterilia) | **-** | **-** | **-** | **-** |
| 164 | 6L1A (mycelia sterilia) | **+** | **++** | **+** | **+** |
| 165 | 8S2 (pycnidium) | **-** | **-** | **-** | **-** |
| 166 | QS1 (pycnidium) | **+** | **-** | **-** | **-** |
| 167 | QS3 (pycnidium) | **-** | **-** | **-** | **-** |
| 168 | FS1 (pycnidium) | **-** | **-** | **-** | **-** |
| 169 | 10L2B (pycnidium) | **-** | **-** | **-** | **-** |
| 170 | 11L1 (pycnidium) | **-** | **-** | **-** | **-** |

*– Not active; ± slightly active; + active; ++ very active.
